# Neutrophil-to-lymphocyte ratio and all-cause mortality with and without myeloproliferative neoplasms—a Danish longitudinal study

**DOI:** 10.1038/s41408-024-00994-z

**Published:** 2024-02-09

**Authors:** Morten Kranker Larsen, Vibe Skov, Lasse Kjær, Christina Schjellerup Eickhardt-Dalbøge, Trine Alma Knudsen, Marie Hvelplund Kristiansen, Anders Lindholm Sørensen, Troels Wienecke, Morten Andersen, Johnny T. Ottesen, Johanne Gudmand-Høyer, Jordan Andrew Snyder, Mikkel Porsborg Andersen, Christian Torp-Pedersen, Henrik Enghusen Poulsen, Thomas Stiehl, Hans Carl Hasselbalch, Christina Ellervik

**Affiliations:** 1 Department of Hematology, Zealand University Hospital, Roskilde, Denmark; 2 Department of Clinical Medicine, Faculty of Health and Medical Sciences, University of Copenhagen, Copenhagen, Denmark; 3 Department of Neurology, Zealand University Hospital, Roskilde, Denmark; 4 Department of Science and Environment, Roskilde University, Roskilde, Denmark; 5 Department of Cardiology, Copenhagen University Hospital, Nordsjællands Hospital, Hillerød, Denmark; 6 Department of Endocrinology, Copenhagen University Hospital, Bispebjerg Frederiksberg Hospital, Copenhagen, Denmark; 7 Institute for Computational Biomedicine - Disease Modelling, Faculty of Medicine, RWTH Aachen University, Aachen, Germany; 8 Department of Clinical Biochemistry, Zealand University Hospital, Koege, Denmark; 9 Department of Laboratory Medicine, Boston Children’s Hospital, Harvard Medical School, Boston, MA USA

**Keywords:** Myeloproliferative disease, Risk factors

## Abstract

The neutrophil-to-lymphocyte ratio(NLR) is increased in chronic inflammation and myeloproliferative neoplasms (MPN). We hypothesize that NLR is associated with all-cause mortality and mortality by comorbidity burden in the general population and individuals with MPN. We included 835,430 individuals from The Danish General Suburban Population Study, general practitioners, and outpatient clinics. We investigated NLR on mortality stratified by prevalent and incident MPN, essential thrombocythemia (ET), polycythemia vera (PV), myelofibrosis (MF), comorbidity burden (CCI-score), and the Triple-A risk score using hazard ratio (HR) and 95% confidence interval (95%CI). NLR 1–1.9 was the reference level. During a median follow-up of 11.2 years, 197,802 deaths were recorded. All-cause mortality increased for a stepwise increasing NLR with a HR (95%CI) for NLR ≥ 6 of 2.06(2.03–2.09) for the whole population and 2.93(2.44–3.50) in prevalent MPN. ET, PV, and MF had a HR (95%CI) for NLR ≥ 2 of 2.14(1.71–2.69), 2.19(1.89–2.54), and 2.31(1.91–2.80). Results were similar for incident MPN. Mortality was higher for stepwise increasing NLR and CCI-score(*p*_*interaction*_ < 2×10^–16^), with a HR for NLR ≥ 6 of 2.23(2.17–2.29), 4.10(4.01–4.20), and 7.69(7.50–7.89), for CCI-score 0, 1–2, or ≥3. The Triple-A risk score demonstrated alignment with NLR. Increasing NLR and comorbidity burden were associated with lower survival in individuals without MPN but were even worse in prevalent and incident MPN, ET, PV, and MF.

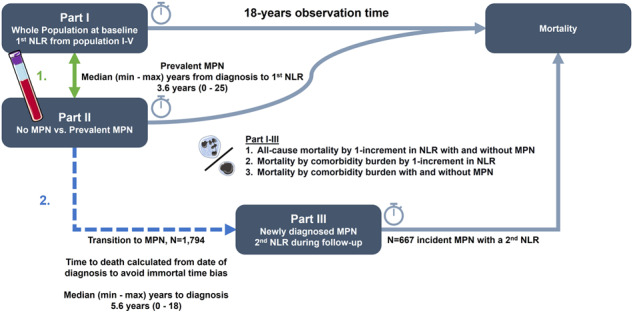

## Introduction

The neutrophil-to-lymphocyte ratio (NLR) is a biomarker that encapsulates chronic inflammation and immunity [1–3]. Elevated NLR is associated with cardiovascular disease [4, 5], autoimmune disease [6–8], cancer [9, 10], and all-cause mortality in the general population [11, 12].

The Philadelphia chromosome-negative classical myeloproliferative neoplasms (MPN) cover essential thrombocythemia (ET), polycythemia vera (PV), and myelofibrosis (MF) [13, 14]. MPN represents a heterogeneous group of acquired hematopoietic stem cell diseases with clonal proliferation of one or more myeloid cell lineages in addition to the clonal makers as the *JAK2V617F*, *CALR*, and *MPL* mutations [15–17]. MPN is associated with an elevated comorbidity burden [18, 19], particularly vascular complications [19–21], and elevated NLR [22]. Neutrophils appear to express high cellular heterogeneity and plasticity in different inflammation-mediated diseases [23, 24] and circulate in an active state in vivo in MPN [25]. Neutrophils can suppress the cytotoxicity of T-lymphocyte and natural killer (NK) cells, reflecting impaired immunity [26–29], which may enhance the clonal progression, evolution, and immune escape of the malignant clone. Hence, NLR may be a suitable biomarker in the clinic that integrates information beyond the absolute neutrophil and lymphocyte count. However, our knowledge of NLR as a predictor for all-cause mortality and mortality by comorbidity burden in individuals with and without MPN is largely unknown. Therefore, in a Danish longitudinal study comprising 835,430 individuals, we hypothesized if NLR is associated with all-cause mortality and mortality by comorbidity burden in the general population and individuals with MPN.

## Materials and methods

### Study population

From 2010 to 2013, the Danish General Suburban Population Study (GESUS) enrolled 21,205 individuals aged ≥20 years with anthropometric, hematological, and biochemical measurements and a detailed questionnaire [30]. GESUS was approved by the regional ethical committee (SJ-114, SJ-452), the Danish Data Protection Agency (REG-50-2015) and adheres to the Declaration of Helsinki. We included 18,938 individuals from GESUS based on the availability of blood cell counts (Population I). In addition, we identified 816,492 individuals aged ≥20 years and retrieved the first occurring neutrophil and lymphocyte count through the Laboratory Information Management Systems (LIMS) from four major geographical locations in Denmark between 2000 and 2012. The LIMS cohorts consisted of individuals from general practitioner visits (Population II) and outpatient clinics from the Capital Region hospitals, Region Zealand hospitals, and Region North hospitals with no inpatient hospitalization or emergency room visits ±15 days from the first blood sample date (Population III–V). Therefore, all blood count measurements were taken by clinical indication. The total study population covering **835,430** was stratified by population origin, Supplementary Fig. 1. Since all individuals in Denmark receive a unique Civil Personal Register (CPR) number at birth or immigration as recorded in the Danish Civil Registration System [31], we could uniquely identify included individuals across different registries [31].

### Blood cell count measurements

The first occurring blood sample date or participation in GESUS was used with an absolute neutrophil granulocyte (ANC) and absolute lymphocyte count (ALC). Blood cell counts were measured by flow cytometry and impedance using Sysmex XE-2100 (GESUS) or XE-5000 (Sysmex Corporation) or ADVIA 120 Hematology System (Siemens Healthineers, Erlingen, Germany). Biochemical measures included C-reactive protein (CRP). All medical laboratories in Denmark are certified by the DS/EN ISO-15189 with internal and external quality control programs.

The ANC and ALC were categorized based on clinical reference intervals. Neutropenia, normal range neutrophil count, and neutrocytosis were defined as a neutrophil count of <2 × 10^9^/L, ≥2 × 10^9^/L to ≤8.8 × 10^9^/L and >8.8 × 10^9^/L, respectively. Lymphopenia, normal range lymphocyte count, and lymphocytosis were defined as a lymphocyte count of <1.3 × 10^9^/L, ≥1.3 × 10^9^/L to ≤3.5 × 10^9^/L, and >3.5 × 10^9^/L, respectively. Individuals with severe neutropenia (<0.5 × 10^9^/L) or lymphopenia (<0.3 × 10^9^/L) were not included in the analysis (*N* = 1369).

The NLR, as exposure, was calculated as a proxy for chronic inflammation and used on a continuous scale with a 1-increment in NLR, categorized as NLR < 1, 1–1.9, 2–2.9, 3–3.9, 4–4.9, 5–5.9, ≥6 or NLR 1–1.9 and ≥2.

### Covariates

Sex and date of birth were ascertained through the Danish Medical Birth Registry [32] and the Danish Civil Registration System [31] to calculate age at the first blood sample date. The highest attainable education was ascertained through the Danish Education Registry [33] and classified as unknown, primary school, high school, vocational training, bachelor, or higher-level education.

### Medication

Lipid-lowering and antihypertensive medication were used as proxies for dyslipidemia and hypertension using the Anatomical Therapeutic Chemical Classification System (ATC), Supplementary Table 1. Hypertension and dyslipidemia were defined if ≥2 redeemed prescriptions were logged in the Danish National Prescription Registry [34]. The first redeemed prescription date was used to define any use prior to the first occurring blood sample date. Since glucocorticoid use impacts both the ANC and ALC, any redeemed prescription, defined by ATC H02AB, −15 days (early exposure) or −16 to −30 days (late exposure) from the first blood sample date or participation in GESUS was investigated.

### Comorbidities

The comorbidity burden was assessed by the Charlsons Comorbidity Index (CCI) [35, 36], Supplementary Table 2. Comorbidities were retrospectively assessed across 19 major disease categories to calculate the CCI-score using the Danish National Patient Registry (DNPR) [37] and the International Classification of Diseases 8th and 10th editions (ICD8 and ICD10). The first blood sample date or participation in GESUS was used as the index date for a retrospective assessment. CCI was categorized as 0, 1–2, and ≥3. Additional disease entities were retrospectively assessed, including ischemic heart disease (IHD), chronic obstructive pulmonary disease (COPD), cancer, and a composite arterial, and composite venous disease variable, Supplementary Table 3.

### MPN-diagnosis

Information on prevalent MPN disease at the first blood sample date or participation in GESUS was obtained through DNPR, Supplementary Table 4. Incident MPN was defined as an MPN diagnosis received during follow-up among individuals with no MPN at the first blood sample date or participation in GESUS. Both prevalent and incident MPN was defined by ICD8 and ICD10, only from the department of hematology as the primary reason for health care contact (A-diagnosis).

### Outcome

All-cause mortality was ascertained through the Danish Register of Causes of Death [38], with all causes of death included. For the mortality by comorbidity burden, we only included natural causes of death, excluding suicide, accidents, and violence. The mortality by comorbidity burden was defined as any prevalent disease at the first blood sample date or participation in GESUS as measured by the CCI-score but also IHD, COPD, cancer of hematological and non-hematological origin, composite arterial, and composite venous diseases. All-cause mortality by NLR and 1st-occurring incident IHD, COPD, composite arterial, or venous diseases were investigated.

### Statistics

We used R 4.0.3, STATA SE 14.2 (StataCorp. College Station, TX), and GraphPad Prism version 7 (GraphPad inc. La, Jolla, CA, USA). A two-sided *P* value < 0.05 was considered statistically significant. Summary statistics were presented as mean and standard deviation (SD). Pearson’s Chi-squared tests were used for categorical variables. Unpaired Student´s *t* test with Welch´s correction or ANOVA test was used for continuous variables. If unequal variance was observed by Levene´s test, the Kruskal-Wallis test was used. Adjusted NLR mean with a 95% confidence interval (95%CI) was investigated according to MPN and CCI score by multiple linear regression analysis. In addition, the ANC and ALC were assessed across NLR groups. We investigated the association between NLR and all-cause mortality using the Kaplan–Meier curve with accompanying logrank-test and survival probabilities using 5-, 10-, 15-, and 18-year follow-up periods. Cox proportional hazard regression models were used to obtain multivariable-adjusted hazard ratio (HR (95%CI)) for NLR groups and on a continuous scale with increments of 1. All-cause mortality was assessed on the whole population with and without nearest neighboring propensity-score matching (PSM) using 0.1 SD in distance in a 1:1 ratio to equalize confounding variables for each NLR group compared to NLR 1–1.9. All-cause mortality across NLR groups was stratified with and without MPN, ET, PV, and MF, whereas mortality by comorbidity burden was stratified by NLR groups and the CCI-score with and without MPN. We applied the −2 log-likelihood ratio test to assess interaction. We performed a meta-analysis to account for population heterogeneity. Random-effect model using DerSimonian and Laird was applied to estimate a pooled effect size in HR (95%CI) for each NLR group across population I–V. Heterogeneity was investigated by I^2^-statistical analyses. During follow-up, we investigated all-cause mortality and mortality by comorbidity burden among individuals in transition to MPN (*N* = 1794). The availability of a 2nd ANC and ALC from the LIMS within a week, month, or year prior to diagnosis defined inclusion (*N* = 667). Individuals with no MPN included at baseline were applied during follow-up if a 2nd ANC and ALC were measured on the date of incident MPN diagnosis. The date of incident MPN diagnosis or 2nd NLR defined a new index date during follow-up. The Triple-A risk score [39] was calculated based on age, ANC, and ALC. Age >70 or age 50–70 was given 4 and 2 points, respectively. ALC < 1.7 × 10^9^/L or ANC ≥ 8 × 10^9^/L was each given 1 point, resulting in the following risk categories: low (0–1 points), intermediate-1 (2–3 points), intermediate-2 (4 points) or high risk (5–6 points). We investigated the association between the Triple-A risk score, NLR, and all-cause mortality in the whole population and MPN. All analyses at baseline (1st NLR) and follow-up (2nd NLR) were adjusted for potential confounders, including age, sex, CCI, antihypertensive medication, lipid-lowering medication, glucocorticoid use, population origin, CRP level, and education. NLR 1–1.9 was used as the reference level for all analyses, Supplementary Figs. 2, 3.

## Results

### Baseline characteristics, NLR, and CCI-score in MPN

**Fig. 1:**
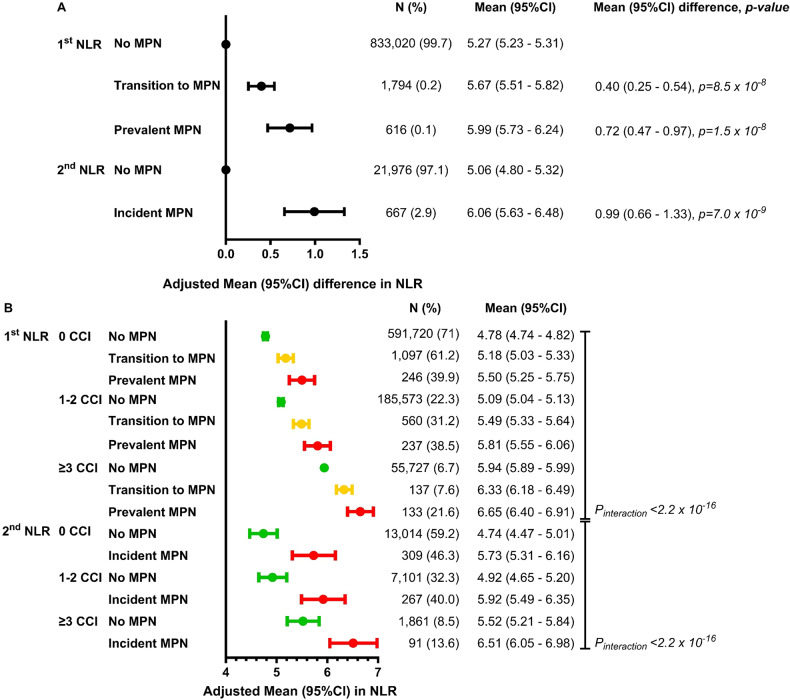
Mean (95%CI) in NLR by MPN and CCI-score. Adjusted mean (95%CI) in NLR comparing individuals with no MPN vs. transition to MPN vs. prevalent MPN stratified by an increase in CCI-score (0 CCI - ≥3 CCI) at baseline (1st NLR). During follow-up, individuals in the transition to MPN (1st NLR) were diagnosed (2^nd^ NLR). Plot **A** was stratified by MPN, and plot **B** was stratified by MPN and CCI score at the 1st and 2nd NLR. The multiple-adjusted linear regression analysis was adjusted for age, sex, population origin, hypertension, hyperlipidemia, CCI score, glucocorticoid use, CRP level, and education. Green colors represent no MPN. Yellow colors represent transition to MPN. Red colors represent prevalent or incident MPN. We tested if MPN and the CCI score interacted on an increase in NLR at the 1st and 2nd NLR. CCI Charlson comorbidity index score. NLR Neutrophil-to-lymphocyte ratio. Prevalent MPN Individuals with myeloproliferative neoplasm at the 1st NLR. Transition to MPN: Individuals with no MPN at the 1st NLR but diagnosed with incident MPN.

At baseline, we included 835,430, of whom 1794 were in transition to MPN, and 616 had prevalent MPN. Individuals with transition to and prevalent MPN were older and had more comorbidities at the 1st NLR (Table 1). NLR was higher in individuals with transition to and prevalent MPN than no MPN with a mean (95%CI) difference of 0.40 (0.25–0.54), *p* = 8.5 × 10^–8^ and 0.72 (0.47–0.97), *p* = 1.5 × 10^–8^, respectively (Fig. 1A), and further increased by the CCI-score (Fig. 1B, *p*_*interaction*_ < 2.2 × 10^–16^). For incident MPN, median time to diagnosis and 2nd NLR was 5.6 years (range: 0–18) from the 1st NLR. Incident MPN had comparable NLR as prevalent MPN. Additional information by NLR, population origin and CCI are shown in Supplementary Tables 5–9 and Supplementary Fig. 4.

**Table 1:** Baseline characteristics (1st NLR).

	No MPN		Transition to MPN^a^		Prevalent MPN		*p value*
	*N*	% / Mean (SD)	*N*	% / Mean (SD)	*N*	% / Mean (SD)	
Sex							
Female	471,929	56.7	1042	58.1	354	57.5	0.4
Male	361,091	43.3	752	41.9	262	42.5	
Age	833,020	51.4 (18.8)	1794	61.4 (14.4)	616	68.3 (13.8)	<2.2 × 10^–16^
Education							
Primary school	201,035	24.1	524	29.2	186	30.2	<2.2 × 10^–16^
High school	39,975	4.8	47	2.6	11	1.8	
Vocational training	294,608	35.4	710	39.6	225	36.5	
Bachelor	135,398	16.3	285	15.9	65	10.6	
Higher education	102,396	12.3	132	7.4	51	8.3	
Unknown	59,608	7.2	96	5.4	78	12.7	
Glucocorticoids							
No use	820,581	98.5	1769	98.6	601	97.6	0.3
Early use	8474	1	15	0.8	10	1.6	
Late use	3965	0.5	10	0.6	5	0.8	
Comorbidities							
Hypertension	207,457	24.9	713	39.7	349	56.7	<2.2 × 10^–16^
Hyperlipidemia	71,689	8.6	250	13.9	95	15.4	<2.2 × 10^–16^
IHD	60,607	7.3	198	11	118	19.2	<2.2 × 10^–16^
COPD	29,574	3.6	82	4.6	56	9.1	7.4 × 10^–14^
Arterial disease	74,689	9	273	15.2	187	30.4	<2.2 × 10^–16^
Venous disease	19,541	2.3	69	3.8	53	8.6	<2.2 × 10^–16^
CCI-score							
0	591,720	71	1097	61.1	246	39.9	<2.2 × 10^–16^
1–2	185,573	22.3	560	31.2	237	38.5	
≥3	55,727	6.7	137	7.6	133	21.6	
Blood cell counts							
Neutrophils (x10^9^/L)	833,020	5.1 (3)	1794	6.8 (4.4)	616	7 (5.9)	<2.2 × 10^–16^
Lymphocytes (x10^9^/L)	833,020	2.1 (2)	1794	2.1 (1.4)	616	1.7 (0.9)	<2.2 × 10^–16^
NLR	833,020	3.1 (3.4)	1794	3.9 (3.5)	616	5 (5.4)	<2.2 × 10^–16^
CRP-level							
No CRP	442,750	53.2	1121	62.5	506	82.1	<2.2 × 10^–16^
CRP ≤ 10 mg/L	293,243	35.2	517	28.8	71	11.5	
CRP > 10 mg/L	97,027	11.6	156	8.7	39	6.3	

Early glucocorticoid use was defined as any redeemed prescription −15 prior to the first blood sample date.

Late glucocorticoid use was defined as any redeemed prescription −16 to −30 days prior to the first blood sample date.

Hypertension and hyperlipidemia were defined by antihypertensive or lipid-lowering medication.

The blood sample date was used as the index date for the retrospective assessment of comorbidities.

*CCI* Charlson comorbidity index score, *NLR* Neutrophil-to-lymphocyte ratio, *Prevalent MPN* Individuals with myeloproliferative neoplasm at the 1st NLR, *IHD* Ischemic heart disease, *COPD* Chronic obstructive pulmonary disease, *CRP* C-reactive protein.

^a^Transition to MPN: Individuals with no MPN at the 1st NLR but diagnosed with incident MPN during follow-up.

### NLR and all-cause mortality in the whole population

**Fig. 2:**
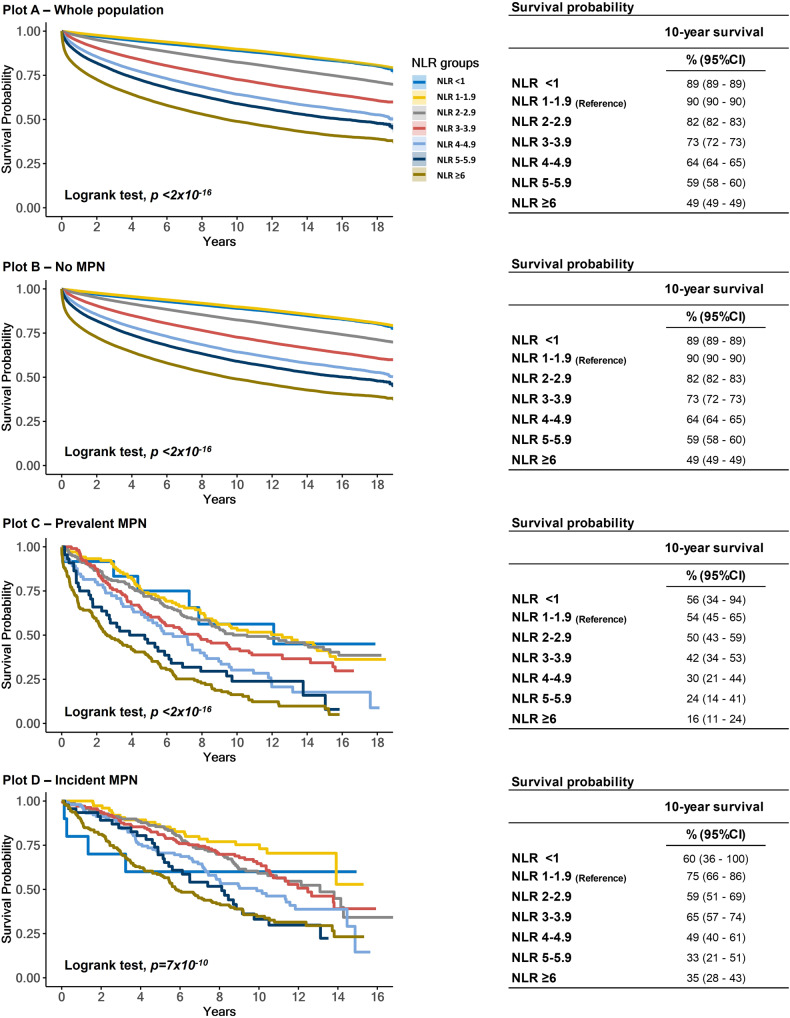
Survival probability by NLR and MPN. Kaplan–Meier curves (plots **A**–**D**) for the whole population, no MPN, prevalent MPN, and incident MPN. Survival probability on the y-axis and years of follow-up since the 1st NLR (plots **A**–**C**) or the 2nd NLR (plot **D**) on the x-axis. The Logrank test was used to compare the survival distribution between NLR groups. When comparing no MPN vs. prevalent MPN (1st NLR), those in transition to MPN were not excluded from the no MPN group. 10-year survival probability was calculated for plots **A**–**D**. NLR Neutrophil-to-lymphocyte ratio.

**Fig. 3:**
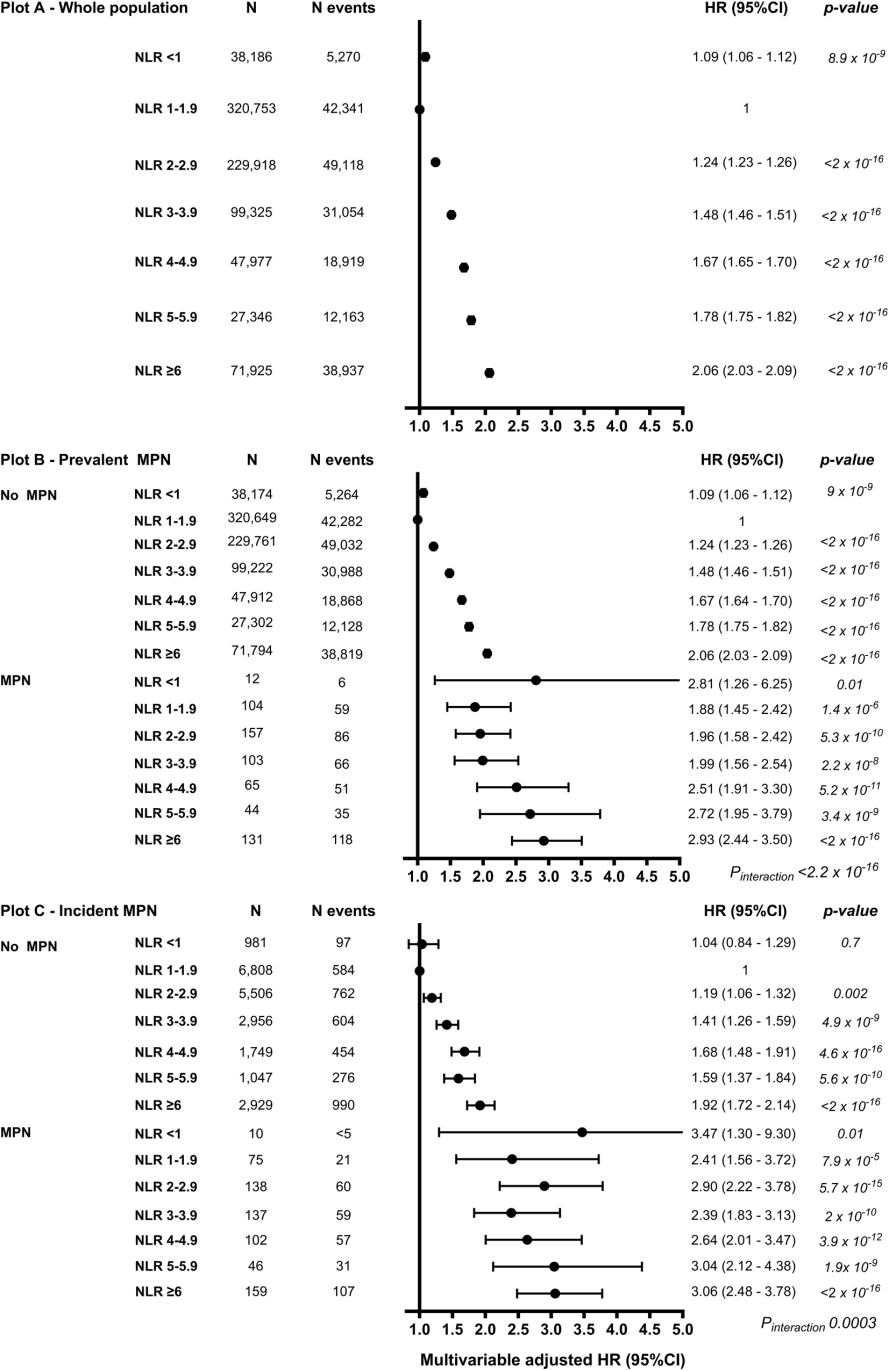
All-cause mortality by NLR and MPN. The multivariable-adjusted Cox regression analysis was adjusted for age, sex, population origin, hypertension, hyperlipidemia, CCI score, glucocorticoid use, CRP level, and education. The NLR 1–1.9 was used as a reference level for plot **A**, whereas NLR 1–1.9 without MPN was used as a reference level in plots **B**, **C**. Plot **B** - Prevalent MPN at baseline (1st NLR). Plot **C** - Incident MPN during follow-up (2nd NLR) contains individuals that developed MPN during the observational period. Using the likelihood ratio test, we tested for interaction between MPN and NLR on all-cause mortality. Survival probabilities for plots **A**–**C**, see Supplementary Table 10. For all-cause mortality by NLR in individuals with prevalent or incident MPN only, see Supplementary Fig. 10.

During a median follow-up of 11.2 years (range: 0–18 years), 197,802 deaths were recorded. For the whole population, a stepwise increase in NLR was associated with a stepwise decrease in 10-year survival probability, with 49% for NLR ≥ 6 compared to 90% for NLR 1–1.9 (Fig. 2A). The ability for NLR to predict all-cause mortality was dependent on the reference interval for both ANC and ALC (Supplementary Fig. 5). In the whole population, increasing NLR was associated with a stepwise increase in all-cause mortality with the highest HR (95%CI) of 2.06 (2.03–2.09), *p* < 2.2 × 10^–16^ among individuals with an NLR ≥ 6 compared to NLR 1–1.9 (Fig. 3A, Supplementary Fig. 6). Results were similar in the PSM models (Supplementary Table 11) and in the meta-analysis for the general population, general practitioners, and hospital cohorts. Notably, NLR < 1 was only associated with all-cause mortality in the hospital cohorts (Supplementary Figs. 7–9).

### NLR and all-cause mortality by MPN

The 10-year survival probability was lower among individuals with prevalent and incident MPN compared to no MPN (Fig. 2B–D). Among individuals with prevalent and incident MPN, we investigated all-cause mortality across the NLR groups compared to individuals with no MPN (Fig. 3B). For all NLR groups, individuals with prevalent MPN (Fig. 3B, *p*_*interaction*_ < 2 × 10^–16^) or incident MPN (Fig. 3C, *p*_*interaction*_ = 0.0003) had a higher HR for all-cause mortality than no MPN. The HR for all-cause mortality with NLR ≥ 6 was 2.93 (2.44–3.50), *p* < 2 × 10^–16^, in individuals with prevalent MPN and 2.06 (2.03–2.09), *p* < 2 × 10^–16^ without MPN. Results were similar for incident MPN. In individuals with prevalent or incident MPN and an NLR 5–5.9 or NLR ≥ 6, the HRs (95%CI) for all-cause mortality were 1.66 (1.18–2.32) and 1.67 (1.28–2.19), respectively, compared to individuals with MPN and an NLR 1–1.9 (Supplementary Fig. 10).

### NLR and all-cause mortality by MPN subtype

**Fig. 4:**
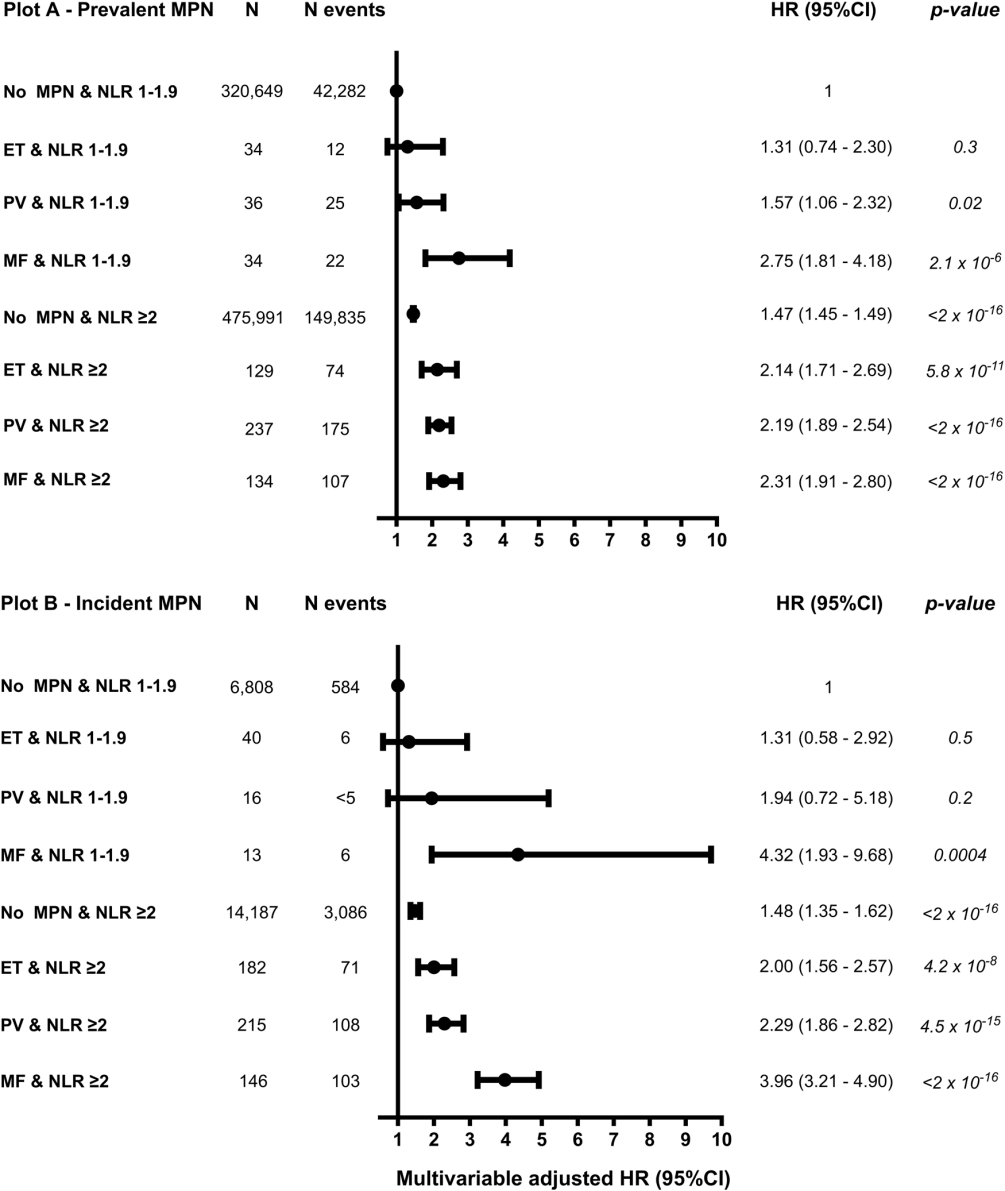
All-cause mortality by NLR and MPN subtype. The multivariable-adjusted Cox regression analysis was adjusted for age, sex, population origin, hypertension, hyperlipidemia, CCI score, glucocorticoid use, CRP level, and education. NLR 1–1.9 without MPN was used as a reference level in both plot **A** and **B**. Plot **A** - Prevalent MPN at baseline (1st NLR). Plot **B** - Incident MPN during follow-up (2nd NLR) contains individuals that developed MPN during the observational period. ET group only contains D473, see Supplementary Fig. 11 with ET defined as D752/D473. For all-cause mortality by MPN subtype only, see Supplementary Fig. 12.

For NLR 1–1.9, individuals with prevalent PV or MF had a HR of 1.57 (1.06–2.32) and 2.75 (1.81–4.18) for all-cause mortality compared to no MPN (Fig. 4A), whereas incident MF had a HR (95%CI) of 4.32 (1.93–9.68) (Fig. 4B). For NLR ≥ 2, individuals with prevalent ET, PV, or MF had a higher HR for all-cause mortality compared to no MPN (Fig. 4A). The HRs for all-cause mortality for prevalent ET, PV, or MF with an NLR ≥ 2 compared to no MPN were 2.14 (1.71–2.69), 2.19 (1.89–2.54), 2.31 (1.91–2.80), and 1.47 (1.45–1.49), respectively. Results were similar for incident ET, PV, and MF, but confidence intervals were wider (Fig. 4B). In individuals with prevalent or incident ET and NLR ≥ 2, the HR (95%CI) for all-cause mortality was 1.64 (1.02–2.62) compared to individuals with ET and an NLR 1–1.9 (Supplementary Fig. 12). The corresponding HR (95%CI) for PV was 1.54 (1.02–2.33).

### NLR and mortality by comorbidity burden

**Fig. 5:**
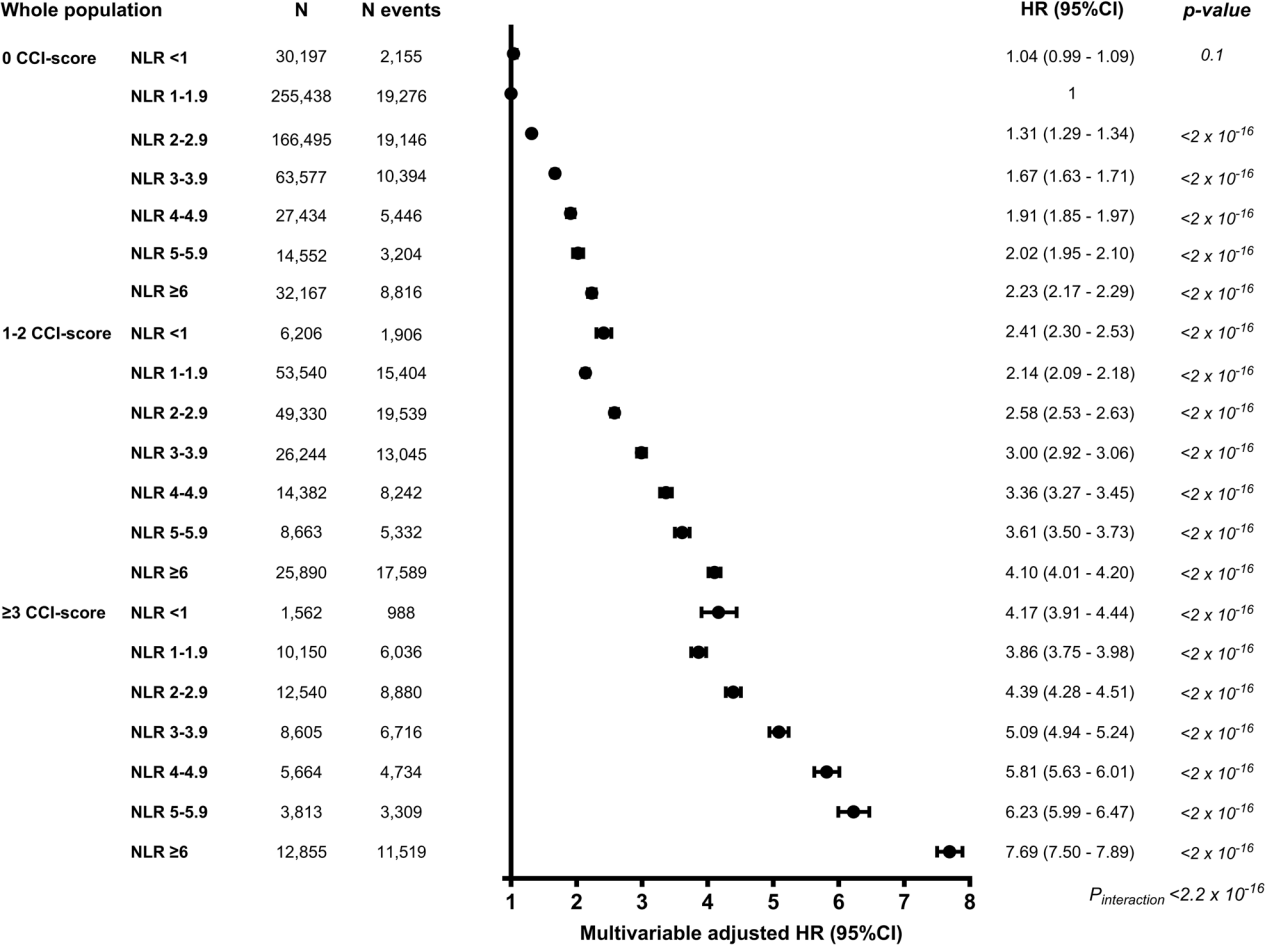
Mortality by comorbidity burden by NLR and CCI-score. The multivariable-adjusted Cox-regression analysis was adjusted for age, sex, population origin, hypertension, hyperlipidemia, glucocorticoid use, CRP level, and education. The NLR 1–1.9 with 0 CCI was used as a reference level in the Cox regression analysis. Using the likelihood ratio test, we tested for interaction between NLR and the CCI score on mortality by comorbidity burden. Only natural causes of death were included (*N* = 191,676). For survival probability, see Supplementary Table 12.

For all NLR groups, individuals with a higher CCI-score had a higher HR for mortality by comorbidity burden compared to no comorbidity (Fig. 5, *p*_*interaction*_ < 2 × 10^–16^). Individuals with NLR ≥ 6 and 0, 1–2, or ≥3 in the CCI-score had a HR (95%CI) of 2.23 (2.17–2.29), 4.10 (4.01–4.20), and 7.69 (7.50–7.89), respectively. A stepwise increase in NLR was also associated with higher mortality by comorbidity burden defined by prevalent or incident IHD, COPD, cancer, composite arterial, and venous diseases (Supplementary Figs. 13, 14, Supplementary Table 13).

### MPN and mortality by comorbidity burden

**Fig. 6:**
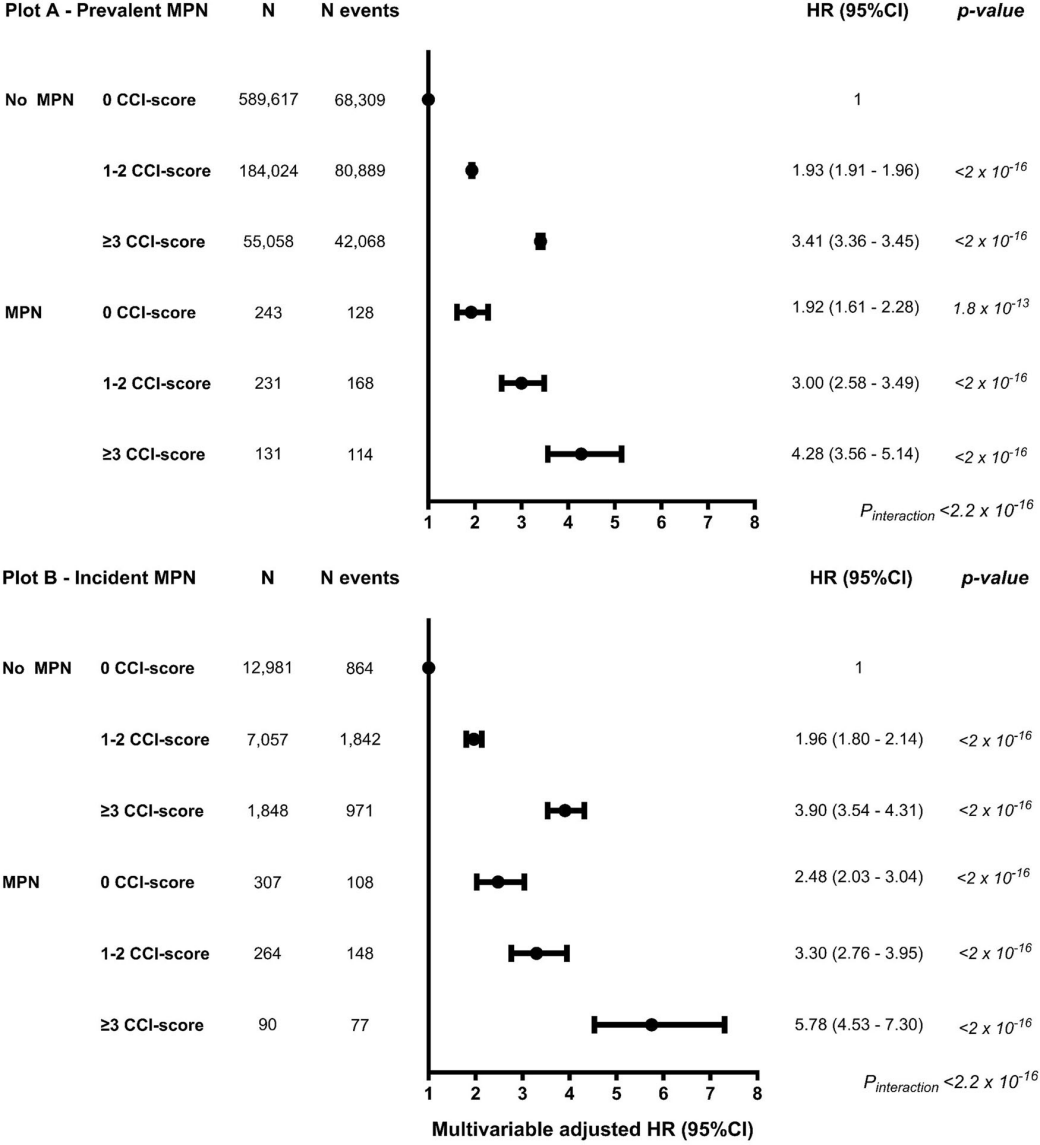
Mortality by comorbidity burden by MPN and CCI-score. The multivariable-adjusted Cox regression analysis was adjusted for age, sex, population origin, hypertension, hyperlipidemia, glucocorticoid use, NLR, CRP level, and education. The No MPN with 0 CCI was used as a reference level in the Cox regression analysis. Plot **A** - Prevalent MPN at baseline (1st NLR). Plot **B** - Incident MPN during follow-up (2nd NLR) contains individuals that developed MPN during the observational period. We tested for interaction between MPN and CCI-score on mortality by comorbidity burden. For survival probability, see Supplementary Table 14.

Higher CCI-score in prevalent MPN (Fig. 6A, *p*_*interaction*_ < 2 × 10^–16^) and incident MPN (Fig. 6B, *p*_*interaction*_ < 2 × 10^–16^) had a higher HR for mortality by comorbidity burden compared to no MPN. Prevalent MPN with 0, 1-2, or ≥3 in the CCI-score compared to no MPN had a HR (95%CI) of 1.92 (1.61–2.28), 3.00 (2.58–3.49), and 4.28 (3.56–5.14). Results were similar for incident MPN (Fig. 6, Supplementary Table 15).

### The Triple-A risk score and all-cause mortality

**Fig. 7:**
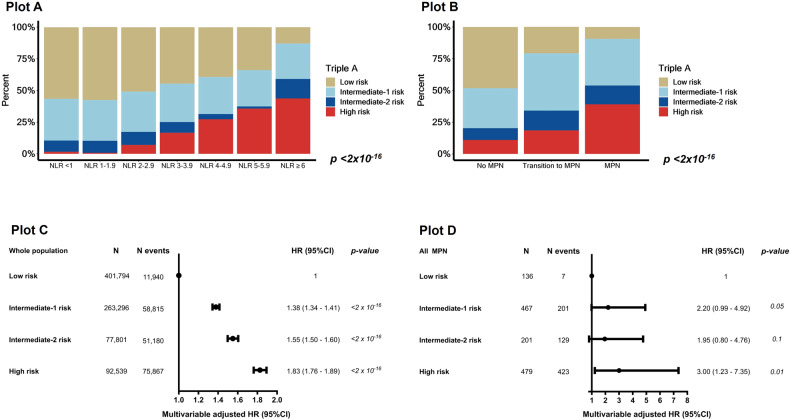
All-cause mortality by the Triple-A risk score. Plot **A**: The absolute percentage of Triple-A risk score categories for each NLR grouping. Plot **B**: The absolute percentage of Triple-A risk score categories among individuals with no MPN, in transition to MPN, and in prevalent MPN. Plot **C**: Multivariable adjusted Cox-regression analysis on the whole population adjusted for age, sex, population origin, hypertension, hyperlipidemia, CCI score, glucocorticoid use, CRP level, and education. Plot **D**: Multivariable adjusted Cox-regression analysis on prevalent and incident MPN, adjusted for age, sex, hypertension, hyperlipidemia, CCI score, glucocorticoid use, CRP level, and education. For unadjusted Triple-A risk score estimates, see Supplementary Table 18. Triple-A risk score - integration of age, absolute lymphocyte count (ALC), and absolute neutrophil count (ANC). Neutrophil-to-lymphocyte ratio.

Individuals with higher Triple-A risk scores were older, had more comorbidities, and had higher NLR (Supplementary Tables 16, 17, Supplementary Fig. 15, Fig. 7A). There was a stepwise increase in the proportion of individuals with the high-risk Triple-A category from no MPN, transitioning to MPN, to prevalent MPN (Fig. 7B). In each Triple-A category, stepwise increasing NLR was associated with higher mortality; this was most pronounced in the high-risk Triple-A category and the least pronounced in the low-risk Triple-A category (Supplementary Fig. 16). Compared to low risk, intermediate-1, intermediate-2, and high risk had a HR (95%CI) of 1.38 (1.34–1.41), 1.55 (1.50–1.60), and 1.83 (1.76–1.89) for all-cause mortality in the whole population. For MPN, only high-risk were associated with mortality with a HR (95%CI) of 3.00 (1.23–7.35) (Fig. 7C, D, Supplementary Tables 18–19).

## Discussion

In this study, individuals with MPN had higher NLR, CCI-score, and Triple-A than those without MPN. Increasing NLR and CCI-score, were associated with lower survival probability in individuals without MPN but were even worse in both prevalent and incident MPN, ET, PV, and MF; these results are novel. There was an interaction between NLR and the CCI-score or MPN on all-cause mortality or mortality by comorbidity burden. Considering only individuals with MPN, NLR was associated with an increased risk of mortality in the highest NLR groups. There was a stepwise increased risk for all-cause mortality by a stepwise increase in the Triple-A category in the whole population, but for MPN, only individuals in the high-risk category had an increased risk of all-cause mortality when adjusting for other residual confounders.

For the whole population, a higher NLR was associated with higher all-cause mortality, like in previous large-scale population studies [11, 12, 40, 41]. Still, an NLR < 1 was also associated with higher all-cause mortality, but only in hospital cohorts. The effect of both NLR and CCI or NLR and MPN exceeded the product of the effect of the two exposures considered separately, which means that the effect of NLR on mortality depends on the values for CCI or the presence of MPN. Thus, having a higher NLR with a higher CCI score or MPN disease indicates an even worse prognosis than considering NLR alone. The ability of NLR to predict all-cause mortality was dependent on whether the ANC and ALC were within or outside the reference intervals. Thus, NLR was dependent on how severe the lymphopenia or neutropenia were. These concepts and limitations are important for the applicability of NLR in a clinical setting.

All-cause mortality was marginally higher for incident MPN with higher NLR or CCI-score than for prevalent MPN. These results may partly be explained by the selection of survivors among individuals with prevalent MPN undergoing cytoreductive treatment compared to newly diagnosed and treatment naïve individuals with MPN. When stratified by MPN subtypes, both prevalent and incident ET or PV with an NLR ≥ 2 had an increased risk of all-cause mortality compared to no MPN with NLR 1–1.9 in the whole population and when compared to ET or PV alone with an NLR 1–1.9, with overlapping confidence intervals. Prevalent and incident MF had an increased risk of all-cause mortality, irrespective of NLR, compared to no MPN with NLR 1–1.9 in the whole population but not compared to MF alone with NLR 1–1.9. Thus, while MF is a more aggressive phenotype than ET or PV, with evidence of chronic inflammation, immune dysregulation, and ineffective hematopoiesis [42], the NLR did not help risk stratify individuals with MF. The observed risk estimates for prevalent MPN, ET, PV, and MF may be attenuated by cytoreductive therapy [22]. However, in this study, we did not have access to information on cytoreductive treatment.

Generally, an increased NLR reflects a hyperproliferative drive of the myeloid innate immune system [43] combined with adaptive immune dysregulation [44–46], which are cardinal manifestations of MPN [15]. Thus, the compartmentalization of the immune system as innate and adaptive is reflected within NLR using the ANC divided by ALC [1, 47]. In MPN, neutrophils circulate in vivo in an active state and demonstrate more inflammatory signaling pathway activation and dysregulated apoptosis [48, 49]. All these molecular deviations are due to the constitutive activation and perturbed JAK-STAT signaling pathway mediated through the oncogenic *JAK2V617F* mutation, being the most prevalent somatic mutation in MPN [16, 17]. Also, during chronic inflammation, the longevity of neutrophils is abnormally prolonged [50]. Neutrophils appear to express high cellular heterogeneity with immunosuppressive potential through the expression of programmed death ligand 1 (PD-L1), arginase-1, or the integrin MAC-1, which in turn impairs the activation of lymphocytes [27, 28, 51, 52]. In addition, NLR, but not the ANC or ALC separately, correlate with reduced NK-cell cytotoxicity in healthy individuals [53].

Both NLR and MPN are associated with several inflammation-mediated diseases [4–12, 18, 19, 22], which are reflected in our study by increasing CCI-score in individuals with prevalent and incident MPN compared to no MPN. Also, NLR correlates with established inflammation biomarkers such as interleukin-6 (IL-6) and CRP [4]. Furthermore, the anti-inflammatory canakinumab, a monoclonal antibody targeted at IL-1beta, which also lowers NLR [4], is currently in a phase-II trial for MF [42]. These observations, combined, make NLR a valuable biomarker for chronic inflammation and impaired immunity.

Individuals in transition to MPN had a lower NLR and Triple-A risk score than prevalent MPN but higher than those who never developed MPN at baseline. At the time of incident MPN diagnosis, the NLR was comparable to the NLR for prevalent MPN. This temporal sequence of results most likely reflects an early and gradual development from Clonal Hematopoiesis of Indeterminate Potential (CHIP) to overt MPN. CHIP is the acquisition of leukemia-associated mutations without evidence of hematological malignancy [54–56]. Our study did not have information on the somatic mutations in the Danish registries. Therefore, our transition to MPN is predetermined, whereas the transition for CHIP to any myeloid malignancy depends on the acquired somatic mutation and other genetic and non-genetic determinants [57, 58].

The Triple-A risk score was originally developed as a novel prognostication tool for individuals with ET [39], but we did not have sufficient power to investigate this group separately. We observed residual confounding for the Triple-A risk score for all confounding variables. Thus, the multivariable-adjusted Triple-A risk estimates for all-cause mortality were lower than the unadjusted risk estimates. In our study, the Triple-A showed alignment with NLR. Also, in the low-risk category with age <50 years, even high NLR was only associated with a very low risk of all-mortality compared to other Triple-A groups. Indicating that the Triple-A is generalizable to the population at large. This study had several strengths and limitations. We included information on glucocorticoid use during the first and second blood sample dates to account for a *falsely* elevated NLR. Since NLR is confounded [59] by acute disease, trauma, cancer, and surgery, we excluded individuals with any inpatient hospitalization or emergency room visits ±15days from the first blood sample date, only allowing individuals with a general practitioner or outpatient clinic visits or participation in a general population study. NLR is likely to exhibit variations and fluctuations contingent upon the MPN stage and during different cytoreductive treatments. Therefore, the 2nd NLR for incident MPN preceded but was in close temporal proximity to the MPN diagnosis to ensure that NLR was not affected by cytoreductive therapy. Although the date of MPN diagnosis can be affected by administrative and diagnostic delays in real life, the first and second blood sample dates were exact. To reduce misclassification bias, we only included individuals with an MPN disease diagnosed at the department of hematology. However, with the limited number of individuals with ET, PV, and MF, we were unable to substantiate the role of the Triple-A risk score comprehensively [39]. Also, to avoid the introduction of collider bias, we applied the whole population stratified with and without MPN [60]. Although we observed population heterogeneity, consistent results were obtained with and without propensity score matching and for each cohort in the meta-analysis.

In conclusion, individuals with MPN had higher NLR and comorbidity burden than individuals with no MPN. Increasing NLR and comorbidity were associated with higher all-cause mortality and mortality by disease burden in individuals without MPN but were even worse in both prevalent and incident MPN, ET, PV, and MF.

### Supplementary information

Supplementary tables and figures

## Data Availability

Due to the European General Data Protection Regulation (GDPR) the dataset cannot be shared publicly. For the NLR Cohort Study: If investigators would like to collaborate, please contact Morten Kranker Larsen. For GESUS: If investigators would like access to data, please contact Dr. Christina Ellervik.

## References

[1] Buonacera A, Stancanelli B, Colaci M, Malatino L (2022). Neutrophil to lymphocyte ratio: an emerging marker of the relationships between the immune system and diseases. Int J Mol Sci.

[2] Furman D, Campisi J, Verdin E, Carrera-Bastos P, Targ S, Franceschi C (2019). Chronic inflammation in the etiology of disease across the life span. Nat Med.

[3] Liu Z, Liang Q, Ren Y, Guo C, Ge X, Wang L (2023). Immunosenescence: molecular mechanisms and diseases. Signal Transduct Target Ther.

[4] Adamstein NH, MacFadyen JG, Rose LM, Glynn RJ, Dey AK, Libby P (2021). The neutrophil-lymphocyte ratio and incident atherosclerotic events: analyses from five contemporary randomized trials. Eur Heart J.

[5] Shah N, Parikh V, Patel N, Patel N, Badheka A, Deshmukh A (2014). Neutrophil lymphocyte ratio significantly improves the Framingham risk score in prediction of coronary heart disease mortality: insights from the National Health and Nutrition Examination Survey-III. Int J Cardiol.

[6] Ma L, Zeng A, Chen B, Chen Y, Zhou R (2019). Neutrophil to lymphocyte ratio and platelet to lymphocyte ratio in patients with systemic lupus erythematosus and their correlation with activity: a meta-analysis. Int Immunopharmacol.

[7] Olsson A, Gustavsen S, Gisselo Lauridsen K, Chenoufi Hasselbalch I, Sellebjerg F, Bach Sondergaard H (2021). Neutrophil-to-lymphocyte ratio and CRP as biomarkers in multiple sclerosis: a systematic review. Acta Neurol Scand.

[8] Jin Z, Cai G, Zhang P, Li X, Yao S, Zhuang L (2021). The value of the neutrophil-to-lymphocyte ratio and platelet-to-lymphocyte ratio as complementary diagnostic tools in the diagnosis of rheumatoid arthritis: a multicenter retrospective study. J Clin Lab Anal.

[9] Cupp MA, Cariolou M, Tzoulaki I, Aune D, Evangelou E, Berlanga-Taylor AJ (2020). Neutrophil to lymphocyte ratio and cancer prognosis: an umbrella review of systematic reviews and meta-analyses of observational studies. BMC Med.

[10] Templeton AJ, McNamara MG, Seruga B, Vera-Badillo FE, Aneja P, Ocana A (2014). Prognostic role of neutrophil-to-lymphocyte ratio in solid tumors: a systematic review and meta-analysis. J Natl Cancer Inst.

[11] Song M, Graubard BI, Rabkin CS, Engels EA (2021). Neutrophil-to-lymphocyte ratio and mortality in the United States general population. Sci Rep.

[12] Fest J, Ruiter TR, Groot Koerkamp B, Rizopoulos D, Ikram MA, van Eijck CHJ (2019). The neutrophil-to-lymphocyte ratio is associated with mortality in the general population: the rotterdam study. Eur J Epidemiol.

[13] Arber DA, Orazi A, Hasserjian R, Thiele J, Borowitz MJ, Le Beau MM (2016). The 2016 revision to the World Health Organization classification of myeloid neoplasms and acute leukemia. Blood.

[14] Arber DA, Orazi A, Hasserjian RP, Borowitz MJ, Calvo KR, Kvasnicka HM (2022). International consensus classification of myeloid neoplasms and acute leukemias: integrating morphologic, clinical, and genomic data. Blood.

[15] Spivak JL (2017). Myeloproliferative neoplasms. N. Engl J Med.

[16] Vainchenker W, Kralovics R (2017). Genetic basis and molecular pathophysiology of classical myeloproliferative neoplasms. Blood.

[17] Luque Paz D, Kralovics R, Skoda RC (2022). Genetic basis and molecular profiling in myeloproliferative neoplasms. Blood.

[18] Hasselbalch HC, Bjorn ME (2015). MPNs as inflammatory diseases: the evidence, consequences, and perspectives. Mediators Inflamm.

[19] Frederiksen H, Szepligeti S, Bak M, Ghanima W, Hasselbalch HC, Christiansen CF (2019). Vascular diseases in patients with chronic myeloproliferative neoplasms - impact of comorbidity. Clin Epidemiol.

[20] Hultcrantz M, Bjorkholm M, Landgren O, Kristinsson SY, Andersson TML (2018). Risk for arterial and venous thrombosis in patients with myeloproliferative neoplasms. Ann Intern Med.

[21] Marchioli R, Finazzi G, Landolfi R, Kutti J, Gisslinger H, Patrono C (2005). Vascular and neoplastic risk in a large cohort of patients with polycythemia vera. J Clin Oncol.

[22] Carobbio A, Vannucchi AM, De Stefano V, Masciulli A, Guglielmelli P, Loscocco GG (2022). Neutrophil-to-lymphocyte ratio is a novel predictor of venous thrombosis in polycythemia vera. Blood Cancer J.

[23] Herrero-Cervera A, Soehnlein O, Kenne E (2022). Neutrophils in chronic inflammatory diseases. Cell Mol Immunol.

[24] Silvestre-Roig C, Braster Q, Ortega-Gomez A, Soehnlein O (2020). Neutrophils as regulators of cardiovascular inflammation. Nat Rev Cardiol.

[25] Hasselbalch HC, Elvers M, Schafer AI (2021). The pathobiology of thrombosis, microvascular disease, and hemorrhage in the myeloproliferative neoplasms. Blood.

[26] Antonucci L, Porcu C, Timperi E, Santini SJ, Iannucci G, Balsano C (2020). Circulating neutrophils of nonalcoholic steatohepatitis patients show an activated phenotype and suppress T lymphocytes activity. J Immunol Res.

[27] Liu K, Huang HH, Yang T, Jiao YM, Zhang C, Song JW (2021). Increased neutrophil aging contributes to t cell immune suppression by PD-L1 and Arginase-1 in HIV-1 treatment naive patients. Front Immunol.

[28] Pillay J, Kamp VM, van Hoffen E, Visser T, Tak T, Lammers JW (2012). A subset of neutrophils in human systemic inflammation inhibits T cell responses through Mac-1. J Clin Investig.

[29] Costantini C, Cassatella MA (2011). The defensive alliance between neutrophils and NK cells as a novel arm of innate immunity. J Leukoc Biol.

[30] Bergholdt HK, Bathum L, Kvetny J, Rasmussen DB, Moldow B, Hoeg T (2013). Study design, participation and characteristics of the danish general suburban population study. Dan Med J.

[31] Schmidt M, Pedersen L, Sorensen HT (2014). The danish civil registration system as a tool in epidemiology. Eur J Epidemiol.

[32] Bliddal M, Broe A, Pottegard A, Olsen J, Langhoff-Roos J (2018). The danish medical birth register. Eur J Epidemiol.

[33] Jensen VM, Rasmussen AW (2011). Danish education registers. Scand J Public Health.

[34] Kildemoes HW, Sorensen HT, Hallas J (2011). The danish national prescription registry. Scand J Public Health.

[35] Quan H, Sundararajan V, Halfon P, Fong A, Burnand B, Luthi JC (2005). Coding algorithms for defining comorbidities in ICD-9-CM and ICD-10 administrative data. Med Care.

[36] Quan H, Li B, Couris CM, Fushimi K, Graham P, Hider P (2011). Updating and validating the Charlson comorbidity index and score for risk adjustment in hospital discharge abstracts using data from 6 countries. Am J Epidemiol.

[37] Lynge E, Sandegaard JL, Rebolj M (2011). The danish national patient register. Scand J Public Health.

[38] Helweg-Larsen K (2011). The danish register of causes of death. Scand J Public Health.

[39] Tefferi A, Loscocco GG, Farrukh F, Szuber N, Mannelli F, Pardanani A (2023). A globally applicable “triple A” risk model for essential thrombocythemia based on age, absolute neutrophil count, and absolute lymphocyte count. Am J Hematol.

[40] Fest J, Ruiter R, Ikram MA, Voortman T, van Eijck CHJ, Stricker BH (2018). Reference values for white blood-cell-based inflammatory markers in the Rotterdam study: a population-based prospective cohort study. Sci Rep..

[41] Azab B, Camacho-Rivera M, Taioli E (2014). Average values and racial differences of neutrophil lymphocyte ratio among a nationally representative sample of United States subjects. PLoS One.

[42] Passamonti F, Mora B (2023). Myelofibrosis. Blood.

[43] Koschmieder S, Mughal TI, Hasselbalch HC, Barosi G, Valent P, Kiladjian JJ (2016). Myeloproliferative neoplasms and inflammation: whether to target the malignant clone or the inflammatory process or both. Leukemia.

[44] Riley CH, Hansen M, Brimnes MK, Hasselbalch HC, Bjerrum OW, Straten PT (2015). Expansion of circulating CD56bright natural killer cells in patients with JAK2-positive chronic myeloproliferative neoplasms during treatment with interferon-alpha. Eur J Haematol.

[45] Riley CH, Jensen MK, Brimnes MK, Hasselbalch HC, Bjerrum OW, Straten PT (2011). Increase in circulating CD4(+)CD25(+)Foxp3(+) T cells in patients with Philadelphia-negative chronic myeloproliferative neoplasms during treatment with IFN-alpha. Blood.

[46] Skov V, Larsen TS, Thomassen M, Riley CH, Jensen MK, Bjerrum OW (2012). Molecular profiling of peripheral blood cells from patients with polycythemia vera and related neoplasms: identification of deregulated genes of significance for inflammation and immune surveillance. Leuk Res.

[47] Zahorec R (2021). Neutrophil-to-lymphocyte ratio, past, present and future perspectives. Bratisl Lek Listy.

[48] Markovic D, Maslovaric I, Djikic D, Cokic VP (2022). Neutrophil death in Myeloproliferative neoplasms: shedding more light on neutrophils as a pathogenic link to chronic inflammation. Int J Mol Sci.

[49] Kiem D, Wagner S, Magnes T, Egle A, Greil R, Melchardt T (2021). The role of neutrophilic granulocytes in philadelphia chromosome negative myeloproliferative neoplasms. Int J Mol Sci.

[50] Silvestre-Roig C, Hidalgo A, Soehnlein O (2016). Neutrophil heterogeneity: implications for homeostasis and pathogenesis. Blood.

[51] Vlkova M, Chovancova Z, Nechvatalova J, Connelly AN, Davis MD, Slanina P (2019). Neutrophil and granulocytic myeloid-derived suppressor cell-mediated T cell suppression significantly contributes to immune dysregulation in common variable immunodeficiency disorders. J Immunol.

[52] Scapini P, Cassatella MA (2014). Social networking of human neutrophils within the immune system. Blood.

[53] Kim BR, Chun S, Cho D, Kim KH (2019). Association of neutrophil-to-lymphocyte ratio and natural killer cell activity revealed by measurement of interferon-gamma levels in a healthy population. J Clin Lab Anal.

[54] Steensma DP (2018). Clinical consequences of clonal hematopoiesis of indeterminate potential. Hematol Am Soc Hematol Educ Program.

[55] Cordua S, Kjaer L, Skov V, Pallisgaard N, Kefala M, Gjerdrum LMR (2021). Early detection of myeloproliferative neoplasms in a Danish general population study. Leukemia.

[56] Cordua S, Kjaer L, Skov V, Pallisgaard N, Hasselbalch HC, Ellervik C (2019). Prevalence and phenotypes of JAK2 V617F and calreticulin mutations in a Danish general population. Blood.

[57] Pich O, Reyes-Salazar I, Gonzalez-Perez A, Lopez-Bigas N (2022). Discovering the drivers of clonal hematopoiesis. Nat Commun.

[58] Kar SP, Quiros PM, Gu M, Jiang T, Mitchell J, Langdon R (2022). Genome-wide analyses of 200,453 individuals yield new insights into the causes and consequences of clonal hematopoiesis. Nat Genet.

[59] Karakonstantis S, Kalemaki D, Tzagkarakis E, Lydakis C (2018). Pitfalls in studies of eosinopenia and neutrophil-to-lymphocyte count ratio. Infect Dis.

[60] Hernan MA, Monge S (2023). Selection bias due to conditioning on a collider. BMJ.

