# Supplementary material for: Neutrophil-to-lymphocyte ratio and all-cause mortality with and without myeloproliferative neoplasms—a Danish longitudinal study
Source: Blood Cancer J. 2024 Feb 9;14(1):28. doi: 10.1038/s41408-024-00994-z (PMC10853217; doi:10.1038/s41408-024-00994-z)
Supplement: Supplementary file 1 — Supplementary tables and figures [file 41408_2024_994_MOESM1_ESM.pdf]

Supplementary tables and figures

Neutrophil-to-lymphocyte ratio and all-cause mortality with and without myeloproliferative neoplasms – A Danish longitudinal study

Short title: NLR, MPN, and All-Cause Mortality

Morten Kranker Larsen[1,2], Vibe Skov[1], Lasse Kjær[1], Christina Schjellerup Eickhardt-Dalbøge[1], Trine Alma Knudsen[1], Marie Hvelplund Kristiansen[2,3], Anders Lindholm Sørensen[1], Troels Wienecke[2,3], Morten Andersen[4], Johnny T. Ottesen[4], Johanne Gudmand-Høyer[4], Jordan Andrew Snyder[4], Mikkel Porsborg Andersen[5], Christian Torp-Pedersen[5], Henrik Enghusen Poulsen[2,5,6]*, Thomas Stiehl[4,7] *, Hans Carl Hasselbalch[1,2]*, Christina Ellervik[2,8,9]*

*Contributed equally

[1]Department of Hematology, Zealand University Hospital, Roskilde, Denmark

[2]Department of Clinical Medicine, Faculty of Health and Medical Sciences, University of Copenhagen, Denmark

[3]Department of Neurology, Zealand University Hospital, Roskilde, Denmark

[4]Department of Science and Environment, Roskilde University, Denmark

[5]Department of Cardiology, Copenhagen University Hospital, Nordsjællands Hospital, Hillerød, Denmark

[6]Department of Endocrinology, Copenhagen University Hospital, Bispebjerg Frederiksberg Hospital, Copenhagen, Denmark

[7]Institute for Computational Biomedicine - Disease Modelling, Faculty of Medicine, RWTH Aachen University, Aachen, Germany

[8]Department of Clinical Biochemistry, Zealand University Hospital, Koege, Denmark

[9]Department of Laboratory Medicine, Boston Children's Hospital, Harvard Medical School, Boston, MA, USA.

Supplementary Figure 1. Flowchart of study population grouped by origin

Supplementary Table 1. Medicine as a proxy marker for dyslipidemia and hypertension

Supplementary Table 2. Charlson Comorbidity Index

Supplementary Table 3. Other disease categories

Supplementary Table 4. Myeloproliferative Neoplasms (MPN) diagnosis

Supplementary Figure 2. Study design of the NLR-Cohort study

Supplementary Figure 3. Flowchart of statistical analysis

Supplementary Table 5. Baseline characteristics by population origin (1st NLR)

Supplementary Table 6. Baseline characteristics by NLR groups (1st NLR)

Supplementary Table 7. CCI-score by MPN (1st NLR)

Supplementary Table 8. CCI-score by population origin (1st NLR)

Supplementary Table 9. CCI-score by NLR groups (1st NLR)

Supplementary Figure 4. Adjusted means (95%CI) for the absolute neutrophil and lymphocyte count across NLR using linear regression analysis

Supplementary Figure 5. Survival probability by NLR across the clinical reference interval for absolute neutrophil and lymphocyte count

Supplementary Table 10. Survival probabilities by NLR and MPN

Supplementary Figure 6. All-cause mortality by 1-increment in NLR

Supplementary Table 11. NLR as an independent predictor of all-cause mortality

Supplementary Figure 7. Meta-analyses of all-cause mortality by each NLR group across population origin

Supplementary Figure 8. Meta-analyses of all-cause mortality by each NLR group across population origin

Supplementary Figure 9. The pooled HR (95%CI) using a random effect model for all-cause mortality by each NLR group across population I-V

Supplementary Figure 10. All-cause mortality by NLR in individuals with prevalent or incident MPN

Supplementary Figure 11. All-cause mortality by NLR and MPN subtype

Supplementary Figure 12. All-cause mortality by NLR and MPN subtype in individuals with prevalent or incident MPN

Supplementary Table 12. Survival probabilities by NLR and CCI

Supplementary Figure 13. Mortality by NLR and major prevalent disease categories

Supplementary Figure 14. Mortality by NLR and 1st occurring major incident disease

Supplementary Table 13. Comorbidities at the time of death by NLR

Supplementary Table 14. Survival probabilities by MPN and CCI-score

Supplementary Table 15. Comorbidities at the time of death by MPN

Supplementary Table 16. Baseline characteristics by the Triple-A risk score (1st NLR)

Supplementary Table 17. CCI-score by the Triple-A risk score (1st NLR)

Supplementary Figure 15. Mean (95%CI) NLR by the Triple-A risk score

Supplementary Figure 16. Survival probability by NLR across the Triple-A risk score

Supplementary Table 18. All-cause mortality by the Triple-A risk score, unadjusted

Supplementary Table 19. Comorbidities at the time of death by the Triple-A risk score

Supplementary Figure 17. Survival probabilities by population origin with total observation time

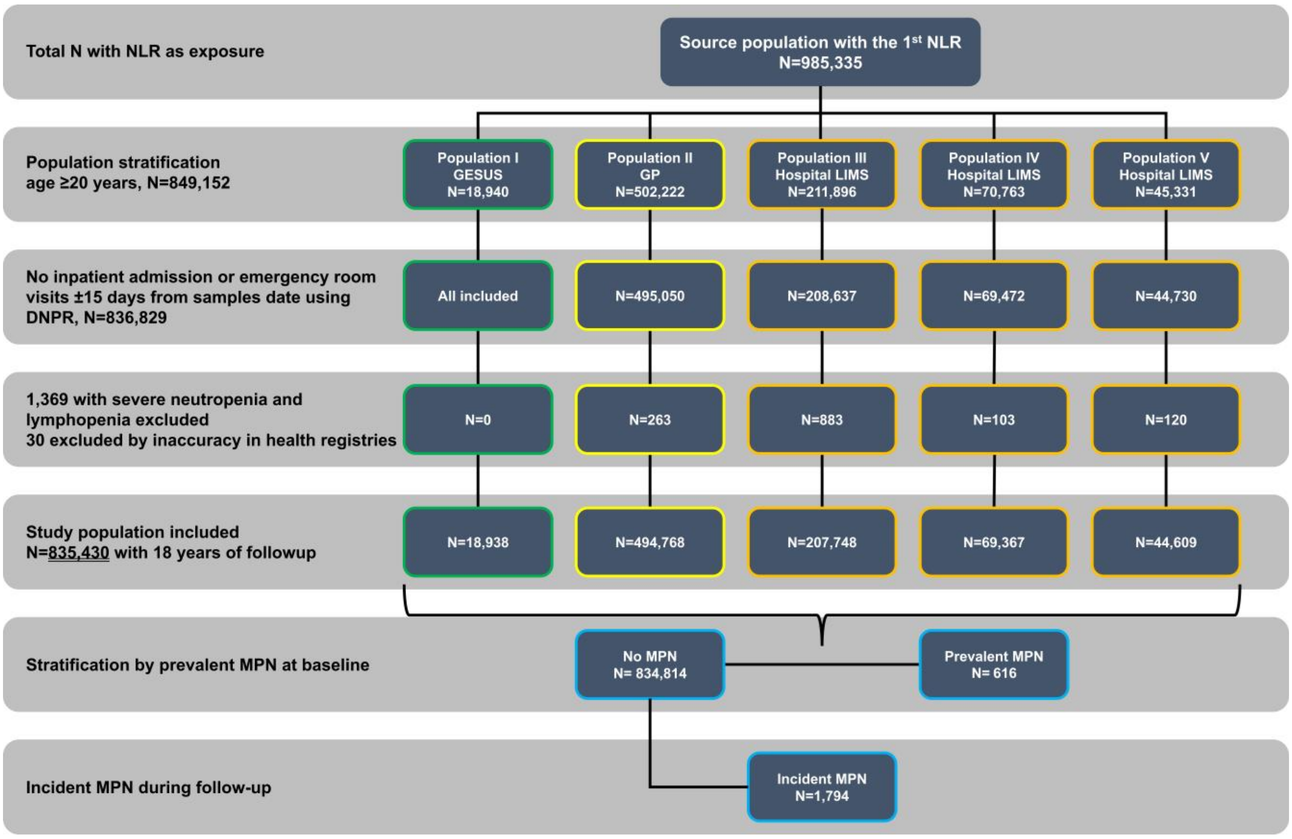

Supplementary Figure 1. Flowchart of study population grouped by origin

The population was grouped into people I-V based on origin.
Population I: The General Suburban Population Study (GESUS) (Green)
Population II: LIMS from General Practitioners (GP) (Yellow)
Population III: LIMS from Capital Region Hospitals – Hospital cohort (Orange)
Population IV: LIMS from Region Zealand Hospitals – Hospital cohort (Orange)
Population V: LIMS from Region North Hospitals – Hospital cohort (Orange)
Abbreviations: NLR: Neutrophil-to-lymphocyte ratio, CRP: C-reactive protein, DNPR: The Danish National Patient Registry, LIMS: Laboratory Information Laboratory System.

Supplementary Table 1. Medicine as a proxy marker for dyslipidemia and hypertension

	ATC
**Lipid-lowering medication**	C10A
HMG-CoA reductase inhibitors	C10AA
Fibrates	C10AB
Bile acid sequestrants	C10AC
Nicotinic acid and derivatives	C10AD
Other lipid-modifying agents	C10AX
**Lipid-lowering medication in combinations**	C10B
Lipid-modifying agents in combination	C10BA
Lipid-modifying agents in combination with other drugs	C10BX*
**Antihypertensive medication**	
Alpha-Adrenoreceptor Antagonist (α-blockers)	C02A, C02B, C02C
Diuretics	C02DA, C03A, C03B, C03D, C03E, C03X, C02L
Agents that inhibit vasoconstriction of arteriolar smooth muscle	C02DB, C02DD, C02DG*
Beta-adrenoceptor antagonists (β-blockers)	C07A, C07B, C07C, C07D, C07F
Calcium antagonist	C08, C08G – in combination with diuretics
Renin-Angiotensin Inhibitors	C09AA
	C09BA – in combination with diuretics
	C09BB – in combination with calcium antagonist
	C09CA
	C09DA – in combination with diuretics
	C09DB – in combination with calcium antagonist
	C09XA02
	C09XA52

All individuals with ≥2 redeemed prescriptions prior to the first blood sample date were defined as having dyslipidemia or hypertension.
*Empty data frame.
Abbreviations: ATC: Anatomical Therapeutic Chemical Classification.

Supplementary Table 2. Charlson Comorbidity Index

Charlson comorbidity categories	ICD8	ICD10
Myocardial infarction	410	I21-I23, I252
Congestive heart failure	42709, 42710, 42711, 42719, 42899, 78249	I099, I110, I130. I132, I255, I420, I425 – I429, P290, I43, I50
Peripheral vascular disease	440 - 445	I70-I72, I731, I738-I739, I77, I790, I792, K551, K558, K559, Z958, Z959
Cerebrovascular disease	430-438	I60-I69, G45, G46, H340
Dementia	290	F00-F03, F051, G30, G311
Chronic pulmonary disease	490-493, 515-518	J40-J47, J60-J67, J684, J84, J701-J703, J920, J953, J961, J982, J983, I278, I279
Connective tissue disease	712, 716, 734, 446, 13599	M05, M06, M08, M09, M30-M36, D86
Ulcer disease	53091, 53098, 531-534	K221, K25-K28
Mild liver disease	571, 57301, 57304	B18, K700-K703, K709, K713-K715, K717, K73, K74, K760, K762-K764, K769, Z944
Moderate to severe liver disease	07000, 07002, 07004, 07006, 07008, 57300, 45601,45609	B150, B160, B162, B190, I850, I859, I864, I982, K704, K711, K721, K729, K765, K766, K767
Diabetes without end-organ damage	24900, 24906, 24907,24909, 25000, 25006, 25007, 25009	E100, E101, E108, E109, E110, E111, E119, E120, E121, E129, E130, E131, E139, E140, E141, E149
Hemiplegia	344	G830-G834, G81-G82, G041, G114, G801-G802, G839
Moderate to severe renal disease	403, 404, 580-584, 59009, 59319, 75310-75319, 792	N032-N037, N052-N057, Z490-Z492, N18 -N19, I120, I131-I132, N250, Z940, Z992, N26
Diabetes with end-organ damage	24901-24905,24908, 25001-25005, 25008	E102 – E107, E112 E113-E118, E122 E123 – E128, E132 E133-E138, E142 E143-E148
Any tumor	140-194 (excl. 173)	C00-C75, C97 (excl. C44)
Leukemia	204-207	C91-C95
Lymphoma	200-203, 275.59	C81-C86, C88, C90, C96
Metastatic solid tumor	195-199	C76-C80
HIV / AIDS	07983	B20-B24

Both A and B diagnoses were included.
Code I132 is included in both the heart failure and the moderate to severe renal disease categories.
Leukemia: Do not include MPN or MDS diagnosis.
Abbreviations: ICD8 & ICD10: International Classification of Diseases 8th & 10th edition

Supplementary Table 3. Other disease categories

	ICD8	ICD10
**Arterial disease**		
Acute myocardial infarction	410	I21-I23, I252
Stroke	431-435	I61, I63, I64, G45
Peripheral artery disease of the lower extremity	440.20	I702
**Venous disease**		
Deep thrombophlebitis	45100, 45108-45109, 45190, 45192, 67101-67103, 67108-67109	I801-I803, O223, O871
Pulmonary embolism	45099, 67399	I260, I269, O882
Splanchnic vein thrombosis	45299, 45301, 45303, 44429, 28944	I81, I820, K550H
**Ischemic Heart Disease**	410-414	I20-I25
**Chronic Obstructive Pulmonary Disease**	491-492	J41-J44
**Cancer**	140-207<br>28729<br>20899<br>209<br>27559	C<br>D45<br>D46<br>D47<br>D752

Both A and B diagnoses were included.
Abbreviations: ICD8 & ICD10: International Classification of Diseases 8th & 10th edition.

Supplementary Table 4. Myeloproliferative Neoplasms (MPN) diagnosis

	ICD8	ICD10
ET	28729	D752 / D473
PV	20899	D459
PMF or sMF	209	D471A, D474A-C
MPN	-	D471
MPN-U	-	D471B

MPN cases were included if defined as A-diagnosis from the department of hematology (medical specialty nr.4). The MF group included MF, MPN, and MPN-U.
Abbreviations: ET: Essential thrombocythemia. PV: Polycythemia vera. P or sMF: Primary of secondary myelofibrosis. MPN-U: Unclassifiable myeloproliferative neoplasms. ICD8 & ICD10: International Classification of Diseases 8th & 10th edition.

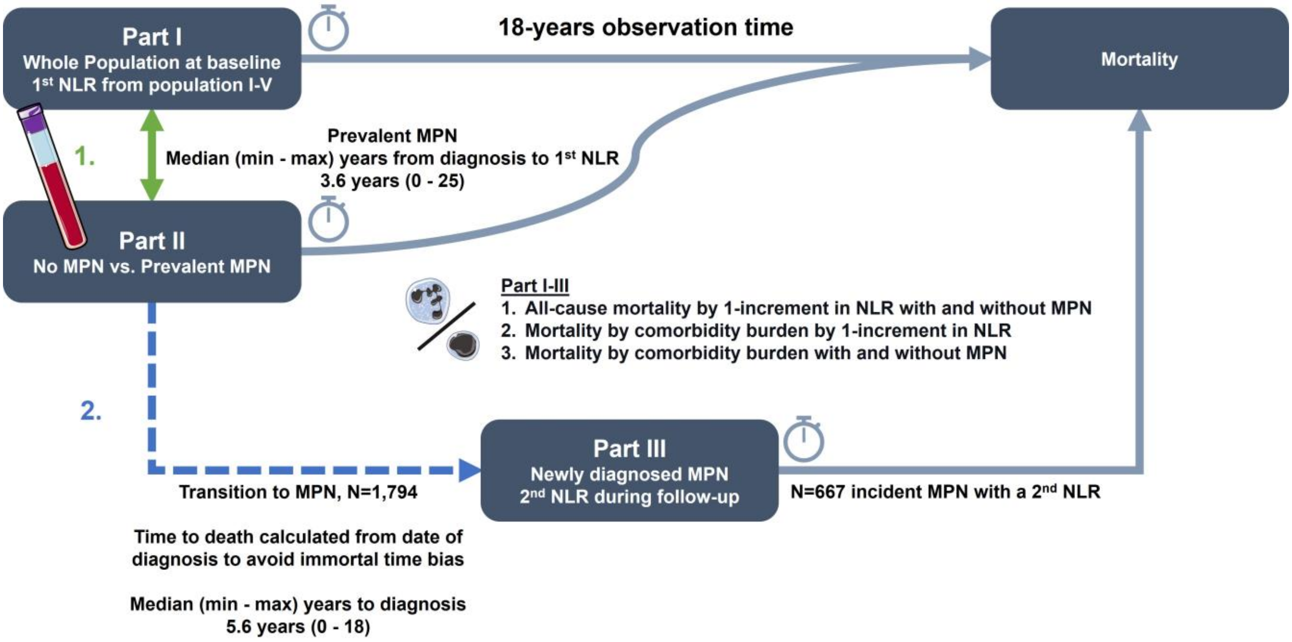

Supplementary Figure 2. Study design of the NLR-Cohort study

This study investigates three large areas relating to the role of NLR on all-cause mortality and mortality by comorbidity burden. **Part I** investigates the role of NLR on mortality for the whole population at baseline (1st NLR). **Part II** investigates the role of NLR on mortality for prevalent MPN compared to no MPN. **Part III** investigates the role of NLR on mortality for incident MPN compared to no MPN during follow-up using a 2nd NLR for individuals included at baseline.

1. For Prevalent MPN: The 1st NLR at baseline. No MPN (N=834,814) compared to prevalent MPN (N=616). When comparing no MPN vs. prevalent MPN (1st NLR), those in transition to MPN were not excluded from the no MPN group.

2. For Incident MPN: During follow-up, 1,794 individuals made the transition from No MPN to MPN. The 2nd NLR within a week, month, or year **prior** to MPN diagnosis compared to a 2nd NLR from individuals that remained in the No MPN group, were alive, and had a blood cell count taken on the exact same date as an MPN diagnosis. Thus, the NLR for incident MPN preceded but was in close temporal proximity to the MPN diagnosis to ensure that NLR was not affected by cytoreductive therapy. Medical Servier Art was used in this figure.

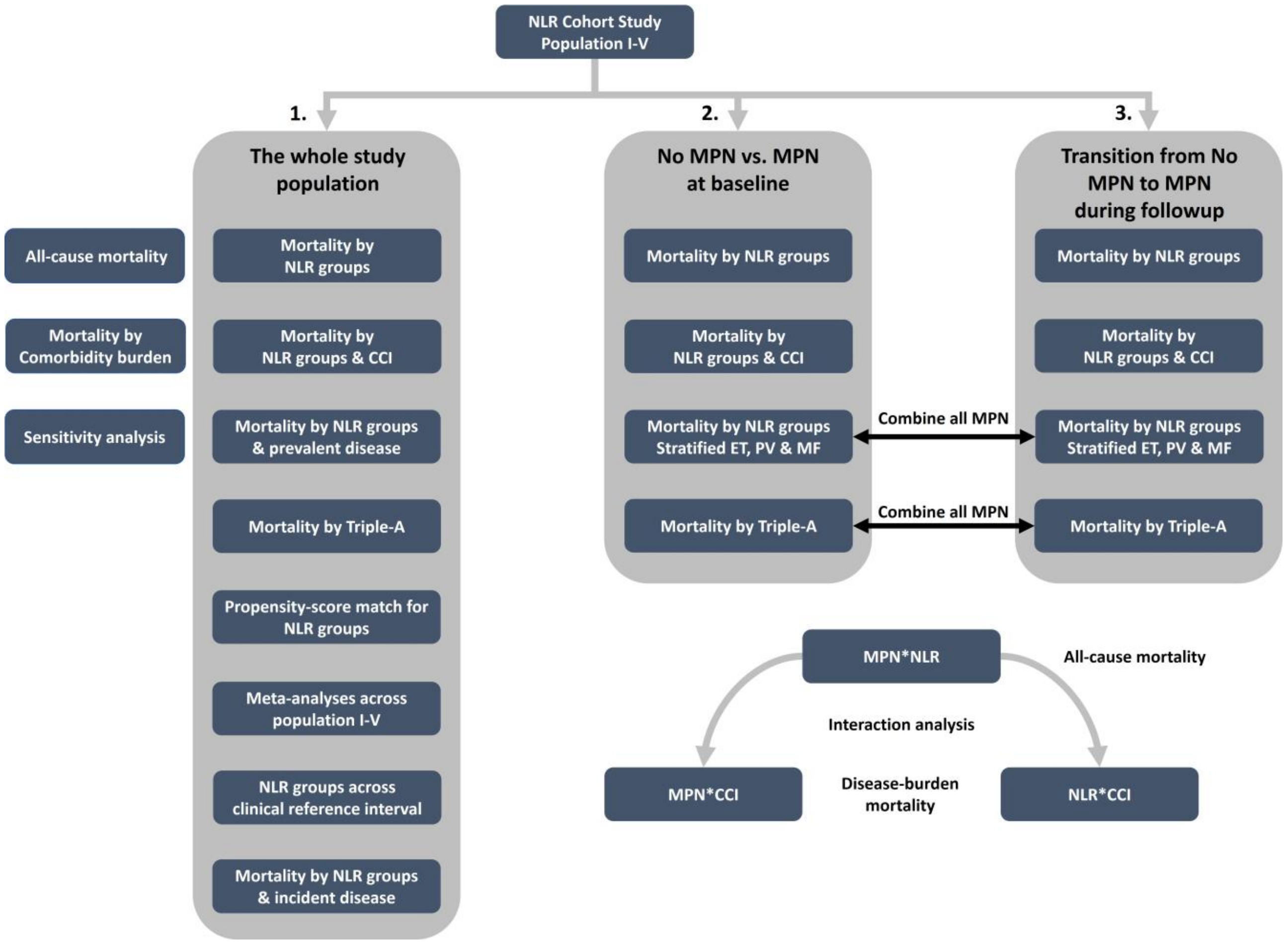

Supplementary Figure 3. Flowchart of statistical analysis

Column 1: Statistical analysis performed on the whole study population stratified by NLR in all-cause mortality and mortality by comorbidity burden.
Column 2: Statistical analysis performed on individuals diagnosed with MPN prior to the first occurring blood sample date, so-called prevalent MPN. Analysis stratified by NLR and CCI-score
Column 3: Statistical analysis was performed on individuals with newly diagnosed MPN, the so-called incident MPN. This design allows individuals in the no MPN group to develop MPN during the observational period.

Supplementary Table 5. Baseline characteristics by population origin (1st NLR)

	Population I		Population II		Population III		Population IV		Population V		
	N	% / Mean(SD)	N	% / Mean(SD)	N	% / Mean(SD)	N	% / Mean(SD)	N	% / Mean(SD)	*p-value*
**Sex**											
Female	10,252	54.1	285,517	57.7	114,775	55.2	39,458	56.9	23,323	52.3	<2.2x10^{-16}
Male	8,686	45.9	209,251	42.3	92,973	44.8	29,909	43.1	21,286	47.7	
**Age**	18,938	56.3 (13.5)	494,768	47.7 (18.3)	207,748	58.1 (18.6)	69,367	53.3 (17.4)	44,609	56.1 (18.1)	<2.2x10^{-16}
**Education**											
Primary school	4,319	22.8	104,361	21.1	58,514	28.2	18,327	26.4	16,224	36.4	
High school	488	2.6	28,357	5.7	8,012	3.9	2,279	3.3	897	2	
Vocational training	9,362	49.4	161,926	32.7	78,671	37.9	28,954	41.7	16,630	37.3	
Bachelor	3,739	19.7	87,039	17.6	28,568	13.8	10,709	15.4	5,693	12.8	<2.2x10^{-16}
Higher education	847	4.5	76,815	15.5	17,790	8.6	5,017	7.2	2,110	4.7	
Unknown	183	1	36,270	7.3	16,193	7.8	4,081	5.9	3,055	6.8	
**Glucocorticoids**											
No use	18,839	99.5	489,792	99	203,067	97.7	67,475	97.3	43,778	98.1	
Early use	60	0.3	3,542	0.7	3,097	1.5	1,233	1.8	567	1.3	<2.2x10^{-16}
Late use	39	0.2	1,434	0.3	1,584	0.8	659	1	264	0.6	
**Comorbidities**											
Hypertension	5,223	27.6	91,747	18.5	79,376	38.2	18,144	26.2	14,029	31.4	<2.2x10^{-16}
Hyperlipidemia	3,262	17.2	24,801	5	33,384	16.1	5,967	8.6	4,620	10.4	<2.2x10^{-16}
IHD	1,294	6.8	21,297	4.3	28,067	13.5	5,591	8.1	4,674	10.5	<2.2x10^{-16}
COPD	443	2.3	10,630	2.1	13,403	6.5	3,254	4.7	1,982	4.4	<2.2x10^{-16}
Arterial disease	1,050	5.5	25,238	5.1	37,045	17.8	6,409	9.2	5,407	12.1	<2.2x10^{-16}
Venous disease	419	2.2	7,414	1.5	9,576	4.6	1,415	2	839	1.9	<2.2x10^{-16}
Cancer	977	5.2	19,172	3.9	23,619	11.4	5,120	7.4	2,722	6.1	<2.2x10^{-16}
CCI-score											
0	14,841	78.4	403,288	81.5	98,650	47.5	46,846	67.5	29,438	66	
1-2	3,493	18.4	74,550	15.1	79,646	38.3	16,603	23.9	12,078	27.1	<2.2x10^{-16}
≥3	604	3.2	16,930	3.4	29,452	14.2	5,918	8.5	3,093	6.9	
**Blood cell counts**											
Neutrophil (x10^{9}/L)	18,938	4.1 (1.3)	494,768	4.5 (2.2)	207,748	6.4 (4)	69,367	5.4 (3.2)	44,609	5.9 (3.9)	<2.2x10^{-16}
Lymphocyte (x10^{9}/L)	18,938	2.3 (0.7)	494,768	2.1 (1.6)	207,748	2 (2.7)	69,367	2.2 (2.8)	44,609	2.1 (1.5)	<2.2x10^{-16}
CRP-level											
No CRP	743	3.9	244,743	49.5	127,796	61.5	39,059	56.3	32,036	71.8	
CRP ≤10mg/L	17,514	92.5	204,943	41.4	40,635	19.6	22,175	32	8,564	19.2	<2.2x10^{-16}
CRP >10mg/L	681	3.6	45,082	9.1	39,317	18.9	8,133	11.7	4,009	9	

Population I: The General Suburban Population Study (GESUS). Population II: General Practitioners (GP). Population III-V: Capital Region, Region Zealand, and Region North Hospitals.
Early glucocorticoid use was defined as any redeemed prescription -15 prior to the first blood sample date. Late glucocorticoid use was defined as any redeemed prescription -16 to -30 days prior to the first blood sample date. The blood sample date was used as the index date for retrospective assessment of comorbidities. Hypertension and hyperlipidemia – are defined by antihypertensive or lipid-lowering medication.
Abbreviations: NLR: Neutrophil-to-lymphocyte ratio. IHD: Ischemic heart Disease. COPD: Chronic obstructive pulmonary disease. CCI-score: Charlson comorbidity index score. CRP: C-reactive protein

Supplementary Table 6. Baseline characteristics by NLR groups (1st NLR)

	NLR <1		NLR 1-1.9		NLR 2-2.9		NLR 3-3.9		NLR 4-4.9		NLR 5-5.9		NLR ≥6		
	N	% / Mean(SD)	N	% / Mean(SD)	N	% / Mean(SD)	N	% / Mean(SD)	N	% / Mean(SD)	N	% / Mean(SD)	N	% / Mean(SD)	*p-value*
**Sex**															
Female	21,076	55.2	181,851	56.7	134,043	58.3	57,112	57.5	26,956	56.2	14,800	54.1	37,487	52.1	<2.2x10^{-16}
Male	17,110	44.8	138,902	43.3	95,875	41.7	42,213	42.5	21,021	43.8	12,546	45.9	34,438	47.9	
**Age**	38,186	46.2 (17.6)	320,753	47.5 (16.9)	229,918	50.9 (18.1)	99,325	54.2 (19.4)	47,977	56.9 (20.2)	27,346	58.7 (20.5)	71,925	62.6 (20.4)	<2.2x10^{-16}
**Education**															
Primary school	7,961	20.8	70,979	22.1	55,739	24.2	25,804	26	12,933	27	7,657	28	20,672	28.7	
High school	2,135	5.6	17,115	5.3	11,035	4.8	4,399	4.4	1,959	4.1	1,045	3.8	2,345	3.3	
Vocational training	12,852	33.7	114,778	35.8	82,951	36.1	34,994	35.2	16,345	34.1	9,410	34.4	24,213	33.7	
Bachelor	7,125	18.7	57,270	17.9	38,053	16.6	14,808	14.9	6,686	13.9	3,505	12.8	8,301	11.5	<2.2x10^{-16}
Higher education	6,138	16.1	45,380	14.1	27,219	11.8	10,643	10.7	4,810	10	2,511	9.2	5,878	8.2	
Unknown	1,975	5.2	15,231	4.7	14,921	6.5	8,677	8.7	5,244	10.9	3,218	11.8	10,516	14.6	
**Glucocorticoids**															
No use	37,947	99.4	318,526	99.3	227,510	99	97,733	98.4	46,813	97.6	26,423	96.6	67,999	94.5	
Early use	171	0.4	1,487	0.5	1,600	0.7	1,101	1.1	794	1.7	650	2.4	2,696	3.7	<2.2x10^{-16}
Late use	68	0.2	740	0.2	808	0.4	491	0.5	370	0.8	273	1	1,230	1.7	
**Comorbidities**															
Hypertension	6,147	16.1	59,330	18.5	56,617	24.6	29,813	30	16,425	34.2	10,096	36.9	30,091	41.8	<2.2x10^{-16}
Hyperlipidemia	2,243	5.9	21,970	6.8	19,714	8.6	10,080	10.1	5,393	11.2	3,258	11.9	9,376	13	<2.2x10^{-16}
IHD	1,746	4.6	15,595	4.9	15,623	6.8	9,046	9.1	5,323	11.1	3,417	12.5	10,173	14.1	<2.2x10^{-16}
COPD	666	1.7	5,813	1.8	6,884	3.0	4,421	4.5	2,893	6	1,932	7.1	7,103	9.9	<2.2x10^{-16}
Arterial disease	1,856	4.9	17,275	5.4	18,711	8.1	11,677	11.8	6,990	14.6	4,527	16.6	14,113	19.6	<2.2x10^{-16}
Venous disease	600	1.6	5,169	1.6	5,205	2.3	2,941	3.0	1,592	3.3	1,096	4	3,060	4.3	<2.2x10^{-16}
Cancer	1,689	4.4	12,372	3.9	12,677	5.5	7,486	7.5	4,595	9.6	2,975	10.9	9,816	13.6	<2.2x10^{-16}
CCI-score															
0	30,319	79.4	256,456	80.0	167,349	72.8	64,019	64.5	27,672	57.7	14,682	53.7	32,566	45.3	
1-2	6,273	16.4	53,998	16.8	49,857	21.7	26,585	26.8	14,554	30.3	8,795	32.2	26,308	36.6	<2.2x10^{-16}
≥3	1,594	4.2	10,299	3.2	12,712	5.5	8,721	8.8	5,751	12	3,869	14.1	13,051	18.1	
**Blood cell counts**															
Neutrophils (x10^9/L)	38,186	2.5 (1.2)	320,753	3.6 (1.1)	229,918	4.8 (1.4)	99,325	5.9 (1.8)	47,977	6.9 (2.2)	27,346	7.9 (2.8)	71,925	11 (5.2)	<2.2x10^{-16}
Lymphocytes (x10^9/L)	38,186	4 (8.6)	320,753	2.4 (0.7)	229,918	2 (0.6)	99,325	1.7 (0.5)	47,977	1.6 (0.5)	27,346	1.4 (0.5)	71,925	1.1 (0.5)	<2.2x10^{-16}
CRP-level															
No CRP	20,148	52.8	167,913	52.4	122,916	53.5	54,126	54.5	26,086	54.4	14,833	54.2	38,353	53.3	
CRP ≤10mg/L	15,966	41.8	137,827	43	85,785	37.3	29,165	29.4	11,018	23	5,076	18.6	8,994	12.5	<2.2x10^{-16}
CRP >10mg/L	2,072	5.4	15,013	4.7	21,217	9.2	16,034	16.1	10,873	22.7	7,435	27.2	24,578	34.2	

Early glucocorticoid use was defined as any redeemed prescription -15 prior to the first blood sample date. Late glucocorticoid use was defined as any redeemed prescription -16 to -30 days prior to the first blood sample date. The blood sample date was used as the index date for retrospective assessment of comorbidities. Hypertension and hyperlipidemia – defined by antihypertensive or lipid-lowering medication.
Abbreviations: NLR: Neutrophil-to-lymphocyte ratio. IHD: Ischemic heart disease. COPD: Chronic obstructive pulmonary disease. CCI-score: Charlson comorbidity index score. CRP: C-reactive protein

Supplementary Table 7. CCI-score by MPN (1st NLR)

	No MPN	Transition to MPN*	Prevalent MPN	
	N (%)	N (%)	N (%)	*p-value*
**Number of individuals**	833,020 (99.7)	1,794 (0.2)	616 (0.1)	
**Acute myocardial infarction**				
No	802,497 (96.3)	1,684 (93.9)	561 (91.1)	
Yes	30,523 (3.7)	110 (6.1)	55 (8.9)	$< 1x10^{-4}$
**Heart failure**				
No	809,735 (97.2)	1,749 (97.5)	569 (92.4)	
Yes	23,285 (2.8)	45 (2.5)	47 (7.6)	$< 1x10^{-4}$
**Peripheral vascular disease**				
No	813,335 (97.6)	1,721 (95.9)	549 (89.1)	
Yes	19,685 (2.4)	73 (4.1)	67 (10.9)	$< 1x10^{-4}$
**Cerebrovascular disease**				
No	782,323 (93.9)	1,616 (90.1)	485 (78.7)	
Yes	50,697 (6.1)	178 (9.9)	131 (21.3)	$< 1x10^{-4}$
**Dementia**				
No	822,989 (98.8)	1,778 (99.1)	597 (96.9)	
Yes	10,031 (1.2)	16 (0.9)	19 (3.1)	$< 1x10^{-4}$
**Chronic pulmonary disease**				
No	777,985 (93.4)	1,664 (92.8)	546 (88.6)	
Yes	55,035 (6.6)	130 (7.2)	70 (11.4)	$< 1x10^{-4}$
**Rheumatic disease**				
No	810,597 (97.3)	1,733 (96.6)	579 (94.0)	
Yes	22,423 (2.7)	61 (3.4)	37 (6.0)	$< 1x10^{-4}$
**Peptic ulcer disease**				
No	808,744 (97.1)	1,731 (96.5)	565 (91.7)	
Yes	24,276 (2.9)	63 (3.5)	51 (8.3)	$< 1x10^{-4}$
**Mild liver disease**				
No	822,480 (98.7)	1,763 (98.3)	605 (98.2)	
Yes	10,540 (1.3)	31 (1.7)	11 (1.8)	0.11
**Severe liver disease**				
No	830,928 (99.7)	1,788 (99.7)	-	
Yes	2,092 (0.3)	6 (0.3)	**<5**	
**Diabetes without end-organ damage**				
No	803,213 (96.4)	1,725 (96.2)	571 (92.7)	
Yes	29,807 (3.6)	69 (3.8)	45 (7.3)	$< 1x10^{-4}$
**Diabetes with end-organ damage**				
No	816,985 (98.1)	1,752 (97.7)	587 (95.3)	
Yes	16,035 (1.9)	42 (2.3)	29 (4.7)	$< 1x10^{-4}$
**Hemiplegia**				
No	830,624 (99.7)	-	-	
Yes	2,396 (0.3)	**<5**	**<5**	
**Moderate to severe renal disease**				
No	825,548 (99.1)	1,776 (99.0)	606 (98.4)	
Yes	7,472 (0.9)	18 (1.0)	10 (1.6)	0.14
**Any tumor**				
No	772,482 (92.7)	1,645 (91.7)	542 (88.0)	
Yes	60,538 (7.3)	149 (8.3)	74 (12.0)	$< 1x10^{-4}$
**Metastatic solid tumor**				
No	826,010 (99.2)	1,785 (99.5)	608 (98.7)	
Yes	7,010 (0.8)	9 (0.5)	8 (1.3)	0.13
**HIV/AIDS**				
No	832,210 (99.9)	1,794 (100.0)	616 (100.0)	
Yes	810 (0.1)	0 (0.0)	0 (0.0)	0.31
**Leukemia**				
No	831,290 (99.8)	1,781 (99.3)	583 (94.6)	
Yes	1,730 (0.2)	13 (0.7)	33 (5.4)	$< 1x10^{-4}$
**Lymphoma**				
No	828,960 (99.5)	1,786 (99.6)	602 (97.7)	
Yes	4,060 (0.5)	8 (0.4)	14 (2.3)	$< 1x10^{-4}$

Each CCI-score component by no MPN vs. transition MPN vs. prevalent MPN at baseline (1st NLR).
P-value calculated by Pearson´s Chi-squared test.
The blood sample date was used as the index date for retrospective assessment of comorbidities.
Each CCI component includes ICD8 & ICD10 codes, as presented in Supplementary Table 2.
* Transition to MPN: Individuals with no MPN at the 1st NLR but diagnosed with incident MPN during follow-up.
Abbreviations: MPN: Myeloproliferative neoplasms.

Supplementary Table 8. CCI-score by population origin (1st NLR)

	Population I	Population II	Population III	Population IV	Population V	
	N (%)	N (%)	N (%)	N (%)	N (%)	*p-value*
**Number of individuals**	18,938 (2.3)	494,768 (59.2)	207,748 (24.9)	69,367 (8.3)	44,609 (5.3)	
**Acute myocardial infarction**						
No	18,472 (97.5)	484,507 (97.9)	193,027 (92.9)	66,694 (96.1)	42,042 (94.2)	
Yes	466 (2.5)	10,261 (2.1)	14,721 (7.1)	2,673 (3.9)	2,567 (5.8)	$< 1x10^{-4}$
**Heart failure**						
No	18,710 (98.8)	486,387 (98.3)	196,758 (94.7)	66,960 (96.5)	43,238 (96.9)	
Yes	228 (1.2)	8,381 (1.7)	10,990 (5.3)	2,407 (3.5)	1,371 (3.1)	$< 1x10^{-4}$
**Peripheral vascular disease**						
No	18,630 (98.4)	488,367 (98.7)	197,799 (95.2)	67,504 (97.3)	43,305 (97.1)	
Yes	308 (1.6)	6,401 (1.3)	9,949 (4.8)	1,863 (2.7)	1,304 (2.9)	$< 1x10^{-4}$
**Cerebrovascular disease**						
No	18,314 (96.7)	477,465 (96.5)	181,967 (87.6)	65,237 (94.0)	41,441 (92.9)	
Yes	624 (3.3)	17,303 (3.5)	25,781 (12.4)	4,130 (6.0)	3,168 (7.1)	$< 1x10^{-4}$
**Dementia**						
No	18,917 (99.9)	490,289 (99.1)	203,341 (97.9)	68,569 (98.8)	44,248 (99.2)	
Yes	21 (0.1)	4,479 (0.9)	4,407 (2.1)	798 (1.2)	361 (0.8)	$< 1x10^{-4}$
**Chronic pulmonary disease**						
No	18,059 (95.4)	471,060 (95.2)	185,968 (89.5)	63,803 (92.0)	41,305 (92.6)	
Yes	879 (4.6)	23,708 (4.8)	21,780 (10.5)	5,564 (8.0)	3,304 (7.4)	$< 1x10^{-4}$
**Rheumatic disease**						
No	18,399 (97.2)	486,631 (98.4)	197,135 (94.9)	67,031 (96.6)	43,713 (98.0)	
Yes	539 (2.8)	8,137 (1.6)	10,613 (5.1)	2,336 (3.4)	896 (2.0)	$< 1x10^{-4}$
**Peptic ulcer disease**						
No	18,574 (98.1)	484,655 (98.0)	197,687 (95.2)	67,413 (97.2)	42,711 (95.7)	
Yes	364 (1.9)	10,113 (2.0)	10,061 (4.8)	1,954 (2.8)	1,898 (4.3)	$< 1x10^{-4}$
**Mild liver disease**						
No	18,858 (99.6)	490,538 (99.1)	202,693 (97.6)	68,576 (98.9)	44,183 (99.0)	
Yes	80 (0.4)	4,230 (0.9)	5,055 (2.4)	791 (1.1)	426 (1.0)	$< 1x10^{-4}$
**Severe liver disease**						
No	18,916 (99.9)	493,897 (99.8)	206,814 (99.6)	69,189 (99.7)	44,512 (99.8)	
Yes	22 (0.1)	871 (0.2)	934 (0.4)	178 (0.3)	97 (0.2)	$< 1x10^{-4}$
**Diabetes without end-organ damage**						
No	18,429 (97.3)	483,686 (97.8)	194,128 (93.4)	66,545 (95.9)	42,721 (95.8)	
Yes	509 (2.7)	11,082 (2.2)	13,620 (6.6)	2,822 (4.1)	1,888 (4.2)	$< 1x10^{-4}$
**Diabetes with end-organ damage**						
No	18,777 (99.1)	489,381 (98.9)	199,881 (96.2)	67,532 (97.4)	43,753 (98.1)	
Yes	161 (0.9)	5,387 (1.1)	7,867 (3.8)	1,835 (2.6)	856 (1.9)	$< 1x10^{-4}$
**Hemiplegia**						
No	18,897 (99.8)	493,809 (99.8)	206,738 (99.5)	69,133 (99.7)	44,451 (99.6)	
Yes	41 (0.2)	959 (0.2)	1,010 (0.5)	234 (0.3)	158 (0.4)	$< 1x10^{-4}$
**Moderate to severe renal disease**						
No	18,840 (99.5)	492,684 (99.6)	204,058 (98.2)	68,125 (98.2)	44,223 (99.1)	
Yes	98 (0.5)	2,084 (0.4)	3,690 (1.8)	1,242 (1.8)	386 (0.9)	$< 1x10^{-4}$
**Any tumor**						
No	17,865 (94.3)	476,165 (96.2)	176,129 (84.8)	63,254 (91.2)	41,256 (92.5)	
Yes	1073 (5.7)	18,603 (3.8)	31,619 (15.2)	6,113 (8.8)	3,353 (7.5)	$< 1x10^{-4}$
**Metastatic solid tumor**						
No	18,882 (99.7)	493,215 (99.7)	203,720 (98.1)	68,525 (98.8)	44,061 (98.8)	
Yes	56 (0.3)	1,553 (0.3)	4,028 (1.9)	842 (1.2)	548 (1.2)	$< 1x10^{-4}$
**HIV/AIDS**						
No	-	494,302 (99.9)	207,495 (99.9)	69,302 (99.9)	44,586 (99.9)	
Yes	**<5**	466 (0.1)	253 (0.1)	65 (0.1)	23 (0.1)	$< 1x10^{-4}$
**Leukemia**						
No	18,917 (99.9)	494,378 (99.9)	206,648 (99.5)	69,144 (99.7)	44,567 (99.9)	
Yes	21 (0.1)	390 (0.1)	1,100 (0.5)	223 (0.3)	42 (0.1)	$< 1x10^{-4}$
**Lymphoma**						
No	18,872 (99.7)	493,744 (99.8)	205,493 (98.9)	68,727 (99.1)	44,512 (99.8)	
Yes	66 (0.3)	1,024 (0.2)	2,255 (1.1)	640 (0.9)	97 (0.2)	$< 1x10^{-4}$

Each CCI-score component by Population I - V
P-value calculated by Pearson´s Chi-squared test.
The blood sample date was used as the index date for retrospective assessment of comorbidities.
Each CCI component includes ICD8 & ICD10 codes, as presented in Supplementary Table 2.
Abbreviations: Population I: The General Suburban Population Study (GESUS). Population II: General Practitioners (GP). Population III-V: Capital Region, Region Zealand, and Region North Hospitals.

Supplementary Table 9. CCI-score by NLR groups (1st NLR)

	NLR <1	NLR 1-1.9	NLR 2-2.9	NLR 3-3.9	NLR 4-4.9	NLR 5-5.9	NLR ≥6	
	N (%)	N (%)	N (%)	N (%)	N (%)	N (%)	N (%)	*p-value*
**Number of individuals**	38,186 (4.6)	320,753 (38.4)	229,918 (27.5)	99,325 (11.9)	47,977 (5.7)	27,346 (3.3)	71,925 (8.6)	
**Acute myocardial infarction**								
No	37,394 (97.9)	313,582 (97.8)	222,324 (96.7)	94,577 (95.2)	45,135 (94.1)	25,476 (93.2)	66,254 (92.1)	
Yes	792 (2.1)	7,171 (2.2)	7,594 (3.3)	4,748 (4.8)	2,842 (5.9)	1,870 (6.8)	5,671 (7.9)	$< 1x10^{-4}$
**Heart failure**								
No	37,750 (98.9)	316,835 (98.8)	224,745 (97.8)	95,513 (96.2)	45,397 (94.6)	25,583 (93.6)	66,230 (92.1)	
Yes	436 (1.1)	3,918 (1.2)	5,173 (2.2)	3,812 (3.8)	2,580 (5.4)	1,763 (6.4)	5,695 (7.9)	$< 1x10^{-4}$
**Peripheral vascular disease**								
No	37,782 (98.9)	316,429 (98.7)	224,878 (97.8)	96,287 (96.9)	46,008 (95.9)	26,146 (95.6)	68,075 (94.6)	
Yes	404 (1.1)	4,324 (1.3)	5,040 (2.2)	3,038 (3.1)	1,969 (4.1)	1,200 (4.4)	3,850 (5.4)	$< 1x10^{-4}$
**Cerebrovascular disease**								
No	36,952 (96.8)	309,135 (96.4)	217,254 (94.5)	91,501 (92.1)	43,254 (90.2)	24,284 (88.8)	62,044 (86.3)	
Yes	1,234 (3.2)	11,618 (3.6)	12,664 (5.5)	7,824 (7.9)	4,723 (9.8)	3,062 (11.2)	9,881 (13.7)	$< 1x10^{-4}$
**Dementia**								
No	38,015 (99.6)	319,060 (99.5)	227,592 (99.0)	97,672 (98.3)	46,887 (97.7)	26,659 (97.5)	69,479 (96.6)	
Yes	171 (0.4)	1,693 (0.5)	2,326 (1.0)	1,653 (1.7)	1,090 (2.3)	687 (2.5)	2,446 (3.4)	$< 1x10^{-4}$
**Chronic pulmonary disease**								
No	36,403 (95.3)	305,524 (95.3)	215,954 (93.9)	91,726 (92.3)	43,538 (90.7)	24,553 (89.8)	62,497 (86.9)	
Yes	1,783 (4.7)	15,229 (4.7)	13,964 (6.1)	7,599 (7.7)	4,439 (9.3)	2,793 (10.2)	9,428 (13.1)	$< 1x10^{-4}$
**Rheumatic disease**								
No	37,515 (98.2)	314,902 (98.2)	224,060 (97.5)	95,924 (96.6)	46,039 (96.0)	26,170 (95.7)	68,299 (95.0)	
Yes	671 (1.8)	5,851 (1.8)	5,858 (2.5)	3,401 (3.4)	1,938 (4.0)	1,176 (4.3)	3,626 (5.0)	$< 1x10^{-4}$
**Peptic ulcer disease**								
No	37,453 (98.1)	314,470 (98.0)	223,852 (97.4)	95,824 (96.5)	45,920 (95.7)	26,001 (95.1)	67,520 (93.9)	
Yes	733 (1.9)	6,283 (2.0)	6,066 (2.6)	3,501 (3.5)	2,057 (4.3)	1,345 (4.9)	4,405 (6.1)	$< 1x10^{-4}$
**Mild liver disease**								
No	37,631 (98.5)	317,436 (99.0)	227,237 (98.8)	97,963 (98.6)	47,216 (98.4)	26,832 (98.1)	70,533 (98.1)	
Yes	555 (1.5)	3,317 (1.0)	2,681 (1.2)	1,362 (1.4)	761 (1.6)	514 (1.9)	1,392 (1.9)	$< 1x10^{-4}$
**Severe liver disease**								
No	38,098 (99.8)	320,257 (99.8)	229,423 (99.8)	99,018 (99.7)	47,788 (99.6)	27,222 (99.5)	71,522 (99.4)	
Yes	88 (0.2)	496 (0.2)	495 (0.2)	307 (0.3)	189 (0.4)	124 (0.5)	403 (0.6)	$< 1x10^{-4}$
**Diabetes without end-organ damage**								
No	37,309 (97.7)	312,992 (97.6)	222,237 (96.7)	94,830 (95.5)	45,475 (94.8)	25,799 (94.3)	66,867 (93.0)	
Yes	877 (2.3)	7,761 (2.4)	7,681 (3.3)	4,495 (4.5)	2,502 (5.2)	1,547 (5.7)	5,058 (7.0)	$< 1x10^{-4}$
**Diabetes with end-organ damage**								
No	37,817 (99.0)	317,100 (98.9)	225,863 (98.2)	96,722 (97.4)	46,463 (96.8)	26,414 (96.6)	68,945 (95.9)	
Yes	369 (1.0)	3,653 (1.1)	4,055 (1.8)	2,603 (2.6)	1,514 (3.2)	932 (3.4)	2,980 (4.1)	$< 1x10^{-4}$
**Hemiplegia**								
No	38,106 (99.8)	320,071 (99.8)	229,291 (99.7)	98,981 (99.7)	47,804 (99.6)	27,240 (99.6)	71,535 (99.5)	
Yes	80 (0.2)	682 (0.2)	627 (0.3)	344 (0.3)	173 (0.4)	106 (0.4)	390 (0.5)	$< 1x10^{-4}$
**Moderate to severe renal disease**								
No	38,040 (99.6)	319,438 (99.6)	228,262 (99.3)	98,140 (98.8)	47,201 (98.4)	26,798 (98.0)	70,051 (97.4)	
Yes	146 (0.4)	1,315 (0.4)	1,656 (0.7)	1,185 (1.2)	776 (1.6)	548 (2.0)	1,874 (2.6)	$< 1x10^{-4}$

	NLR <1	NLR 1-1.9	NLR 2-2.9	NLR 3-3.9	NLR 4-4.9	NLR 5-5.9	NLR ≥6	
	N (%)	N (%)	N (%)	N (%)	N (%)	N (%)	N (%)	*p-value*
**Any tumor**								
No	36,782 (96.3)	306,899 (95.7)	214,735 (93.4)	89,946 (90.6)	42,302 (88.2)	23,610 (86.3)	60,395 (84.0)	
Yes	1,404 (3.7)	13,854 (4.3)	15,183 (6.6)	9,379 (9.4)	5,675 (11.8)	3,736 (13.7)	11,530 (16.0)	$< 1x10^{-4}$
**Metastatic solid tumor**								
No	38,055 (99.7)	319,594 (99.6)	228,466 (99.4)	98,266 (98.9)	47,258 (98.5)	26,839 (98.1)	69,925 (97.2)	
Yes	131 (0.3)	1,159 (0.4)	1,452 (0.6)	1,059 (1.1)	719 (1.5)	507 (1.9)	2,000 (2.8)	$< 1x10^{-4}$
**HIV/AIDS**								
No	38,081 (99.7)	320,413 (99.9)	229,738 (99.9)	99,253 (99.9)	47,934 (99.9)	27,330 (99.9)	71,871 (99.9)	
Yes	105 (0.3)	340 (0.1)	180 (0.1)	72 (0.1)	43 (0.1)	16 (0.1)	54 (0.1)	$< 1x10^{-4}$
**Leukemia**								
No	37,258 (97.6)	320,429 (99.9)	229,714 (99.9)	99,219 (99.9)	47,930 (99.9)	27,309 (99.9)	71,795 (99.8)	
Yes	928 (2.4)	324 (0.1)	204 (0.1)	106 (0.1)	47 (0.1)	37 (0.1)	130 (0.2)	$< 1x10^{-4}$
**Lymphoma**								
No	37,888 (99.2)	319,760 (99.7)	228,887 (99.6)	98,749 (99.4)	47,608 (99.2)	27,125 (99.2)	71,331 (99.2)	
Yes	298 (0.8)	993 (0.3)	1,031 (0.4)	576 (0.6)	369 (0.8)	221 (0.8)	594 (0.8)	$< 1x10^{-4}$

Each CCI-score component by NLR groups
P-value calculated by Pearson´s Chi-squared test.
The blood sample date was used as the index date for retrospective assessment of comorbidities.
Each CCI component includes ICD8 & ICD10 codes, as presented in Supplementary Table 2.
Abbreviations: NLR: Neutrophil-to-lymphocyte ratio.

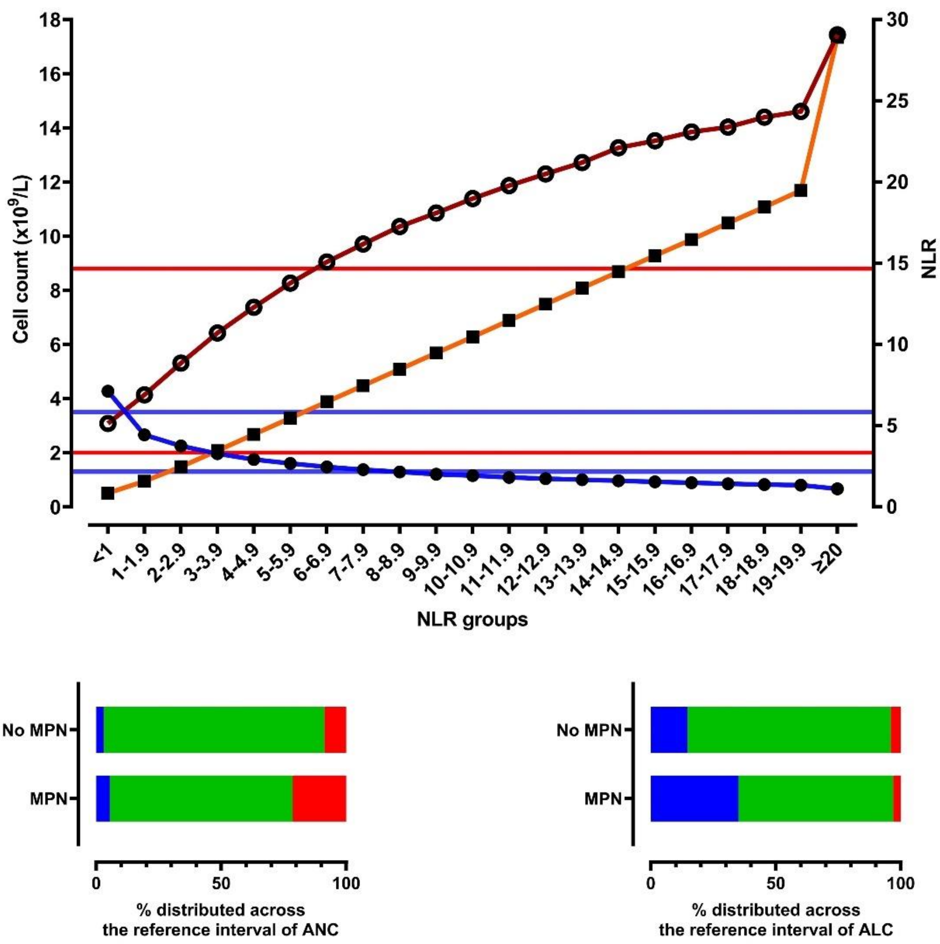

Supplementary Figure 4. Adjusted means (95%CI) for the absolute neutrophil and lymphocyte count across NLR using linear regression analysis

With an increase in ANC (red line with open circle), there is a drop in ALC (blue line with closed circle) and, accordingly, an increase in NLR (orange line with closed square). The red, blue, and orange lines represent a 95% confidence interval (95%CI).

The right Y-axis represents the NLR, whereas the left Y-axis represents cell counts (x10^9/L).

The lower and upper blue lines flank the normal range for ALC: ≥1.3 to ≤ 3.5x109/L, whereas the lower and upper red lines flank the normal range for ANC: ≥2 to ≤ 8.8x10^9/L.

The multiple-adjusted linear regression analysis was adjusted for age, sex, population origin, hypertension, hyperlipidemia, CCI score, glucocorticoid use, CRP level, and education.

Left bar plot: Neutropenia (blue), normal range ANC (green) count, and neutrocytosis (red) were defined as neutrophil count of <2x10^9/L, ≥2x10^9/L to ≤8.8x10^9/L and >8.8x10^9/L, respectively.

Right bar plot: Lymphopenia (blue), normal range ALC (green), and lymphocytosis (red) were defined as a lymphocyte count of <1.3x10^9/L, ≥1.3x10^9/L to ≤3.5x10^9/L, and >3.5x10^9/L, respectively.

Abbreviations: ANC: Absolute neutrophil count. ALC: Absolute lymphocyte count. NLR: Neutrophil-to-lymphocyte ratio.

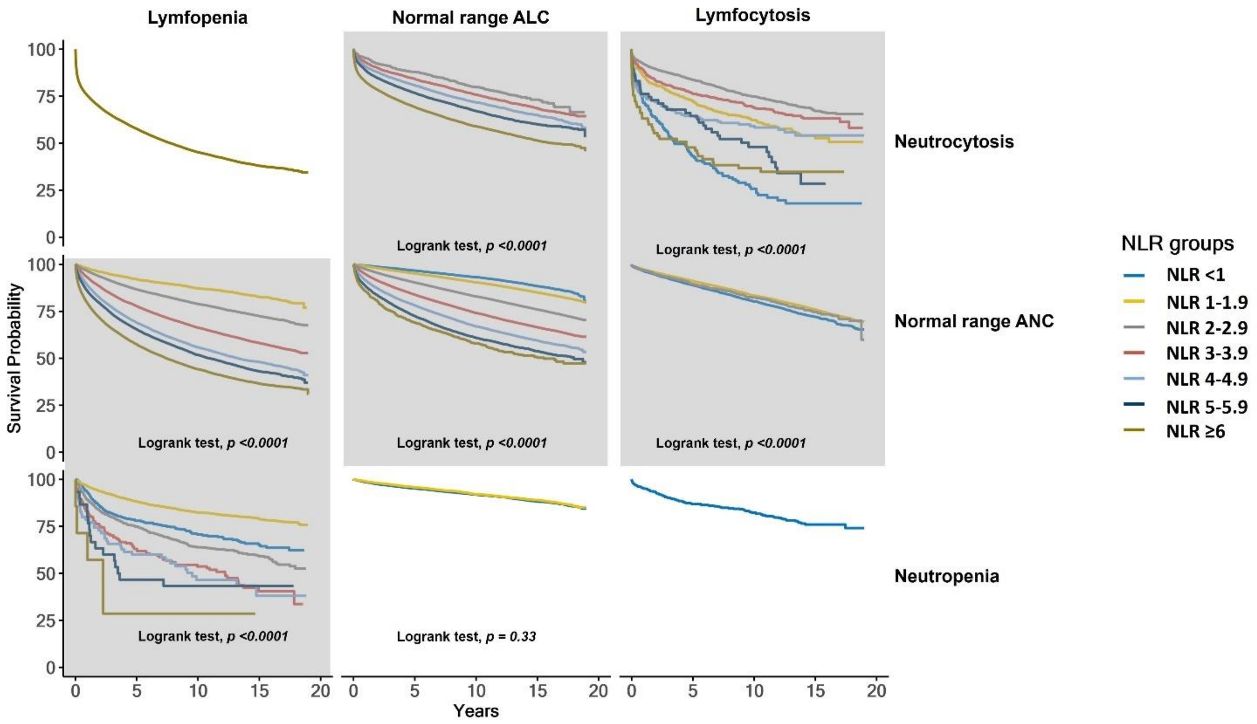

Supplementary Figure 5. Survival probability by NLR across the clinical reference interval for absolute neutrophil and lymphocyte count

Gray-shaded areas represent significant differences between NLR groups.
The Logrank test was used to compare the survival distribution between NLR groups for each matrix.
Neutropenia: <2x10^9/L. The normal range for ANC: ≥ 2 to ≤ 8.8x10^9/L. Neutrocytosis: >8.8x10^9/L.
Lymphopenia: <1.3x10^9/L. The normal range for ALC: ≥1.3 to ≤3.5x10^9/L. Lymphocytosis: >3.5x10^9/L.
Abbreviations: ANC: Absolute neutrophil count. ALC: Absolute lymphocyte count. NLR: Neutrophil-to-lymphocyte ratio.

Supplementary Table 10. Survival probabilities by NLR and MPN

	5-year survival	10-year survival	15-year survival	18-year survival
	% (95%CI)	% (95%CI)	% (95%CI)	% (95%CI)
**Plot A**				
**Whole Population**				
**NLR <1**	94 (94 - 94)	89 (89 - 89)	84 (83 - 84)	80 (80 - 81)
**NLR 1-1.9**	95 (95 - 95)	90 (90 - 90)	84 (84 - 85)	81 (80 - 81)
**NLR 2-2.9**	90 (90 - 90)	82 (82 - 83)	76 (75 - 76)	71 (71 - 72)
**NLR 3-3.9**	83 (82 - 83)	73 (72 - 73)	65 (65 - 65)	61 (60 - 61)
**NLR 4-4.9**	76 (75 - 76)	64 (64 - 65)	57 (56 - 57)	53 (52 - 53)
**NLR 5-5.9**	71 (70 - 71)	59 (58 - 60)	51 (50 - 52)	48 (47 - 49)
**NLR ≥6**	61 (61 - 61)	49 (49 - 49)	41 (41 - 42)	39 (38 - 40)
**Plot B**				
**No MPN**				
**NLR <1**	94 (94 - 94)	89 (89 - 89)	84 (83 - 84)	80 (80 - 81)
**NLR 1-1.9**	95 (95 - 95)	90 (90 - 90)	84 (84 - 85)	81 (80 - 81)
**NLR 2-2.9**	90 (90 - 90)	82 (82 - 83)	76 (75 - 76)	71 (71 - 72)
**NLR 3-3.9**	83 (82 - 83)	73 (72 - 73)	65 (65 - 65)	61 (60 - 61)
**NLR 4-4.9**	76 (75 - 76)	64 (64 - 65)	57 (56 - 57)	53 (52 - 53)
**NLR 5-5.9**	71 (70 - 71)	59 (58 - 60)	51 (50 - 52)	48 (47 - 49)
**NLR ≥6**	61 (61 - 61)	49 (49 - 49)	41 (41 - 42)	39 (39 - 40)
**Prevalent MPN**				
**NLR <1**	75 (54 - 100)	56 (34 - 94)	45 (23 - 89)	-
**NLR 1-1.9**	73 (65 - 82)	54 (45 - 65)	41 (32 - 53)	36 (27 - 49)
**NLR 2-2.9**	72 (65 - 79)	50 (43 - 59)	42 (34 - 52)	39 (30 - 49)
**NLR 3-3.9**	61 (52 - 71)	42 (34 - 53)	34 (25 - 46)	-
**NLR 4-4.9**	58 (48 - 72)	30 (21 - 44)	18 (9.6 - 33)	8.9 (1.9 - 40)
**NLR 5-5.9**	45 (33 - 63)	24 (14 - 41)	16 (6.0 - 42)	-
**NLR ≥6**	37 (30 - 47)	16 (11 - 24)	9.8 (5.5 - 17)	-
**Plot C**				
**No MPN**				
**NLR <1**	96 (95 – 97)	92 (90 – 93)	83 (78 – 89)	-
**NLR 1-1.9**	97 (97 – 98)	93 (92 – 94)	88 (86 – 89)	-
**NLR 2-2.9**	95 (95 – 96)	88 (88 – 89)	79 (77 – 81)	-
**NLR 3-3.9**	91 (90 – 92)	82 (81 – 84)	73 (71 – 76)	-
**NLR 4-4.9**	87 (85 – 88)	77 (75 – 79)	67 (65 – 71)	-
**NLR 5-5.9**	87 (85 – 89)	77 (75 – 80)	65 (61 – 70)	-
**NLR ≥6**	81 (80 – 83)	69 (67 – 71)	57 (54 – 61)	-
**Incident MPN**				
**NLR <1**	60 (36 – 100)	60 (36 – 100)	-	-
**NLR 1-1.9**	85 (78 – 94)	75 (66 – 86)	53 (29 – 95)	-
**NLR 2-2.9**	86 (80 – 92)	59 (51 – 69)	34 (21 – 55)	-
**NLR 3-3.9**	81 (75 – 88)	65 (57 – 74)	39 (28 – 55)	-
**NLR 4-4.9**	71 (62 – 80)	49 (40 – 61)	15 (3.2 – 67)	-
**NLR 5-5.9**	70 (57 – 84)	33 (21 – 51)	-	-
**NLR ≥6**	58 (51 – 67)	35 (28 – 43)	23 (15 – 36)	-

Survival probabilities using 5-, 10-, 15-, and 18-year follow-up periods.
For Plot C, we only calculated survival probability until the 15th year, given the shorter observation time.
Correspond to Figure 3 – All-cause mortality by NLR and MPN.

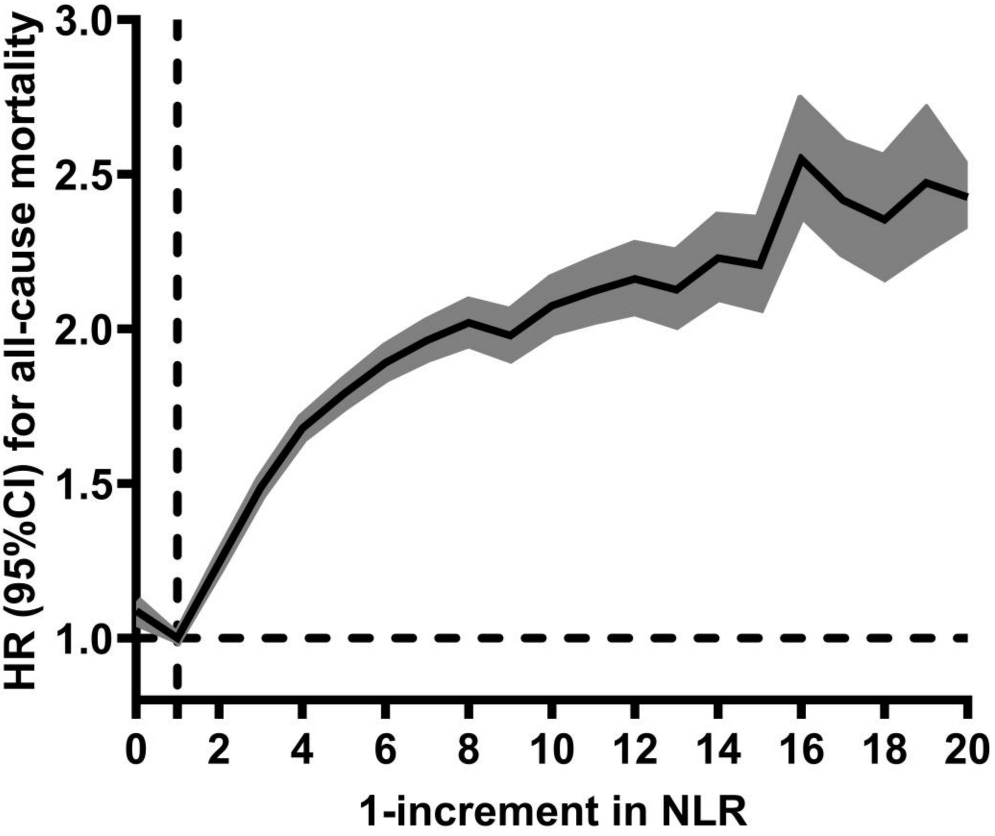

Supplementary Figure 6. All-cause mortality by 1-increment in NLR

The multivariable-adjusted Cox-regression analysis was adjusted for age, sex, population origin, hypertension, hyperlipidemia, CCI score, glucocorticoid use, CRP level, and education.
The Y-axis represents HR (95%CI), whereas the X-axis represents a 1-increment in NLR on a discrete scale from 0-20. The NLR 1-1.9 group was used as a reference level in the Cox-regression analysis.
Abbreviations: NLR: Neutrophil-to-lymphocyte ratio. HR (95%CI): Hazard ratio with 95% confidence interval derived from the Cox proportional hazard regression model.

Supplementary Table 11. NLR as an independent predictor of all-cause mortality

	SMD (Var. Ratio)	N	N events	HR (95%CI)	*p-value*
**1-1.9 NLR (Reference)**	0.000 (1)	38,186	4,736	1	
**<1 NLR**		38,186	5,270	1.14 (1.10 - 1.19)	*$2.2x10^{-11}$*
**1-1.9 NLR (Reference)**	0.025 (1.1)	221,735	37,180	1	
**2-2.9 NLR**		221,735	44,676	1.24 (1.22 - 1.25)	*$<2x10^{-16}$*
**1-1.9 NLR (Reference)**	0.012 (1.05)	96,351	21,929	1	
**3-3.9 NLR**		96,351	29,175	1.43 (1.40 - 1.45)	*$<2x10^{-16}$*
**1-1.9 NLR (Reference)**	0.009 (1.04)	46,694	13,088	1	
**4-4.9 NLR**		46,694	17,959	1.53 (1.50 -1.57)	*$<2x10^{-16}$*
**1-1.9 NLR (Reference)**	0.004 (1.02)	26,627	8,476	1	
**5-5.9 NLR**		26,627	11,586	1.57 (1.53 - 1.62)	*$<2x10^{-16}$*
**1-1.9 NLR (Reference)**	0.03 (1.08)	51,148	15,877	1	
**≥ 6 NLR**		51,148	24,017	1.84 (1.80 - 1.88)	*$<2x10^{-16}$*

Nearest neighboring propensity-score matching (PSM) using 0.1 SD in distance in a 1:1 ratio to equalize confounding variables for each NLR group compared to NLR 1-1.9 as reference. Each PSM model is presented with a standardized mean difference and variance ratio.
Each PSM model included age, sex, population origin, hypertension, hyperlipidemia, CCI score, glucocorticoid use, CRP level, and education.
Abbreviations: NLR: Neutrophil-to-lymphocyte ratio. SMD: Standardized mean difference. Var.ratio: Variance ratio. HR (95%CI): Hazard ratio with 95% confidence interval derived from the Cox proportional hazard regression model.

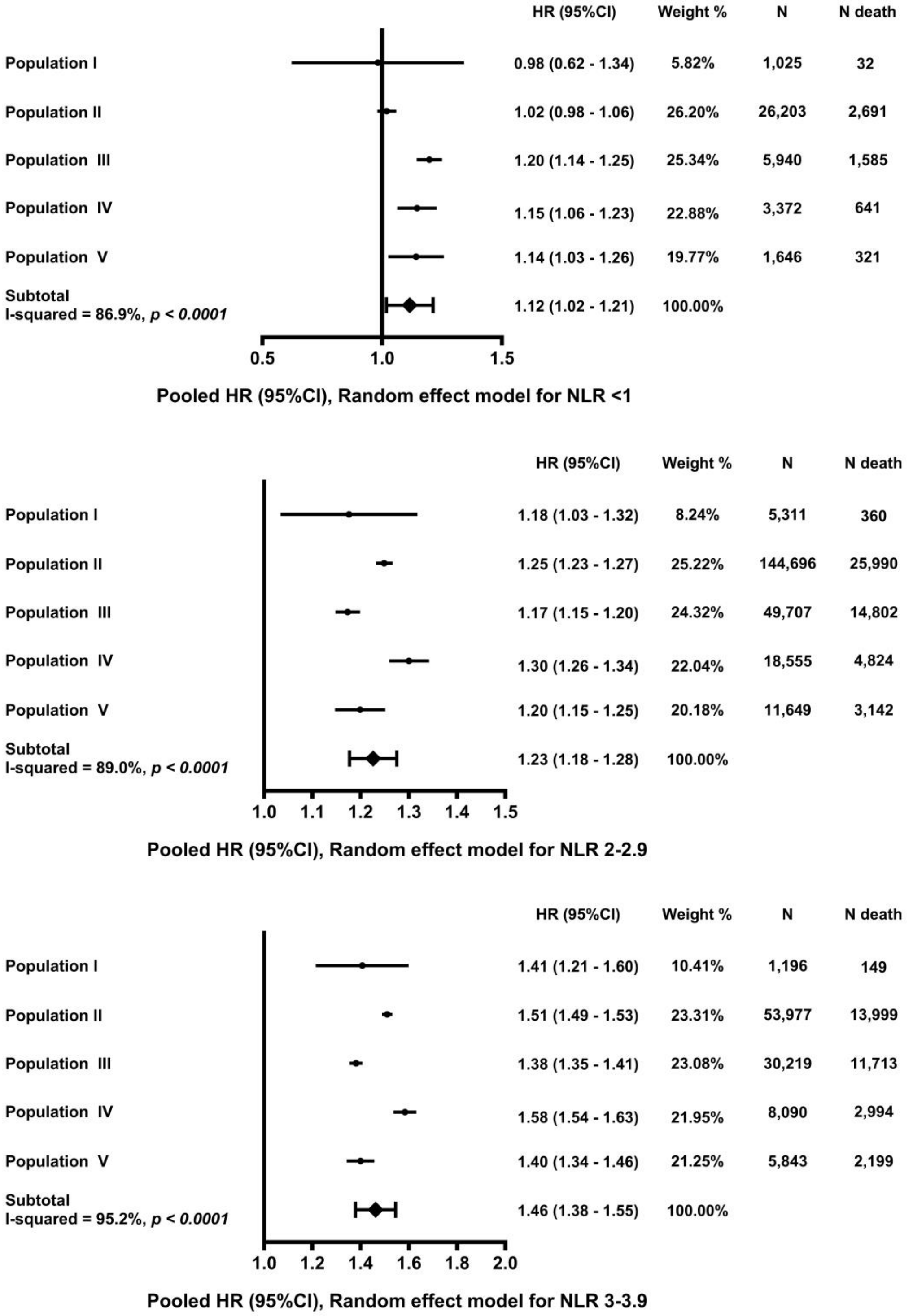

Supplementary Figure 7. Meta-analyses of all-cause mortality by each NLR group across population origin

Each NLR group was compared to NLR 1-1.9. A random effect model was used to calculate the pooled effect size (Hazard ratio with 95% confidence interval (HR (95%CI)). Population heterogeneity was assessed by I^2-squared.
Abbreviations: Population I: The General Suburban Population Study (GESUS). Population II: General Practitioners (GP). Population III-V: Capital Region, Region Zealand, and Region North Hospitals. NLR: Neutrophil-to-lymphocyte ratio.

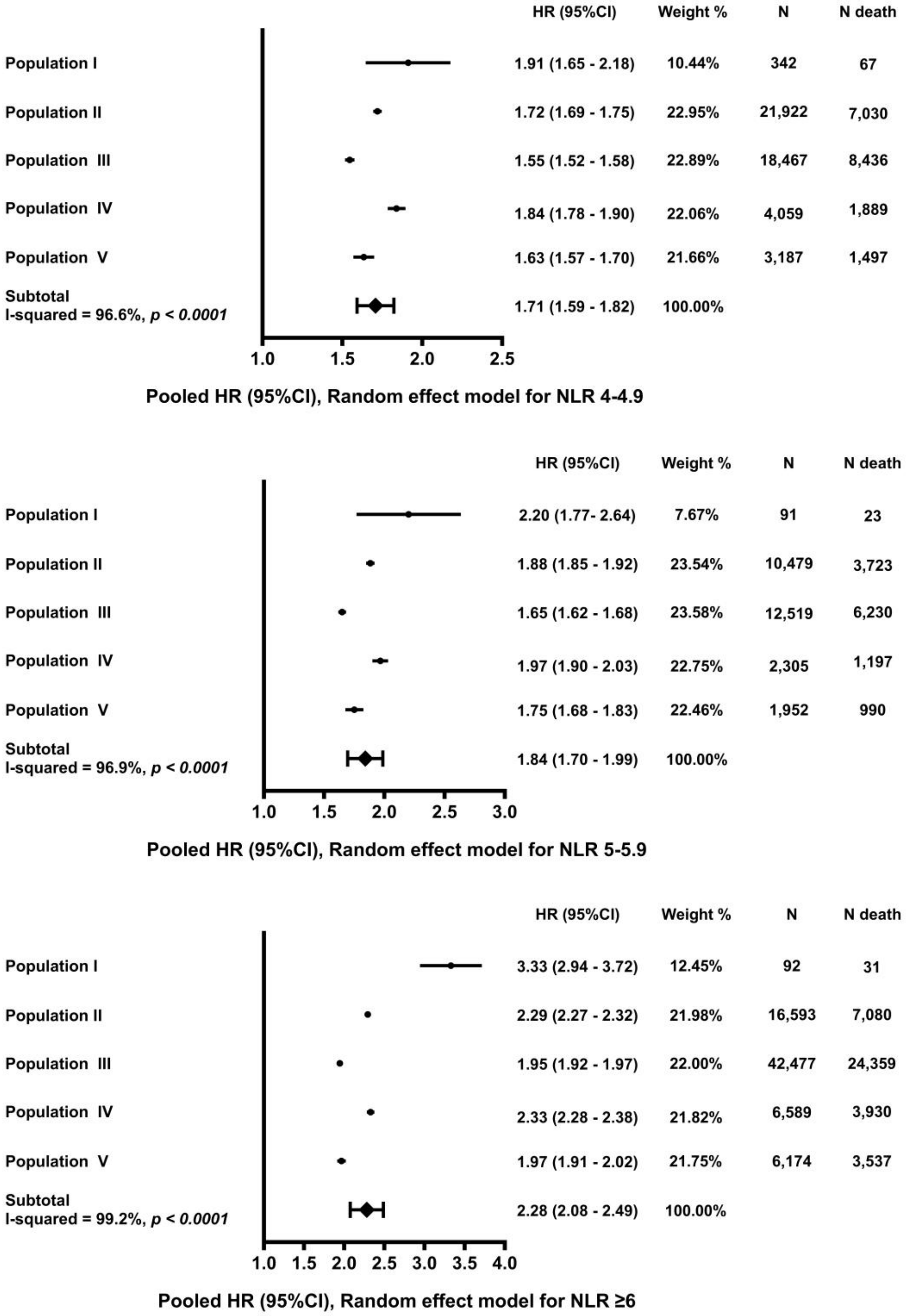

Supplementary Figure 8. Meta-analyses of all-cause mortality by each NLR group across population origin

Each NLR group was compared to NLR 1-1.9. A random effect model was used to calculate the pooled effect size (Hazard ratio with 95% confidence interval (HR (95%CI)). Population heterogeneity was assessed by I^2-squared.

Abbreviations: Population I: The General Suburban Population Study (GESUS). Population II: General Practitioners (GP). Population III-V: Capital Region, Region Zealand, and Region North Hospitals. NLR: Neutrophil-to-lymphocyte ratio.

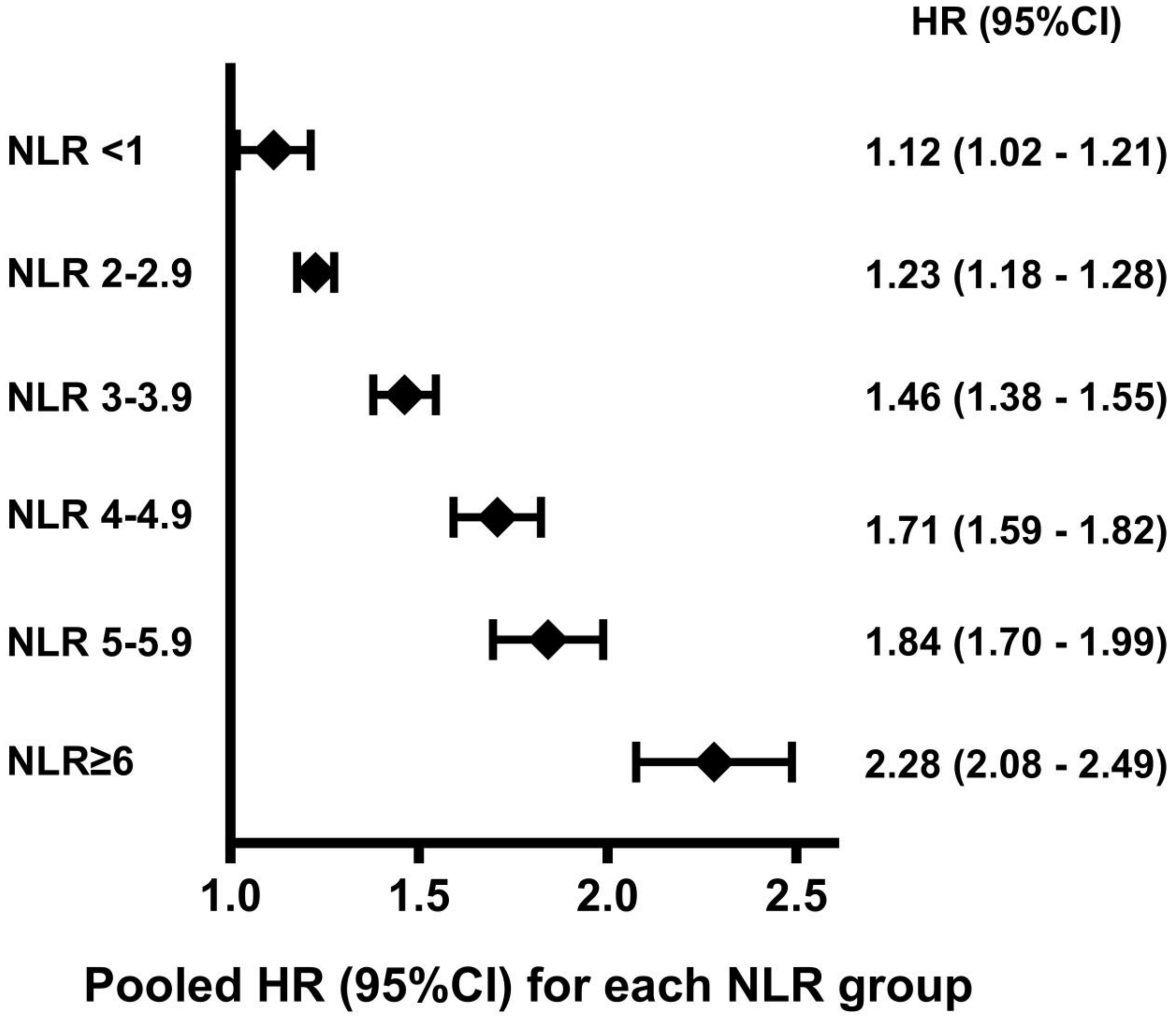

Supplementary Figure 9. The pooled HR (95%CI) using a random effect model for all-cause mortality by each NLR group across population I-V

Each NLR group was compared to NLR 1-1.9. A random effect model was used to calculate the pooled effect size (Hazard ratio with 95% confidence interval (HR (95%CI)).
Abbreviations: NLR: Neutrophil-to-lymphocyte ratio.

All MPN	N	N events	HR (95%CI)	*p-value*
**NLR <1**	22	10	1.40 (0.72 - 2.72)	*0.3*
**NLR 1-1.9**	179	80	1	
**NLR 2-2.9**	295	146	1.15 (0.87 - 1.52)	*0.3*
**NLR 3-3.9**	240	125	1.09 (0.82 - 1.46)	*0.5*
**NLR 4-4.9**	167	108	1.30 (0.96 - 1.75)	*0.08*
**NLR 5-5.9**	90	66	1.66 (1.18 - 2.32)	*0.003*
**NLR ≥6**	290	225	1.67 (1.28 - 2.19)	*0.0002*

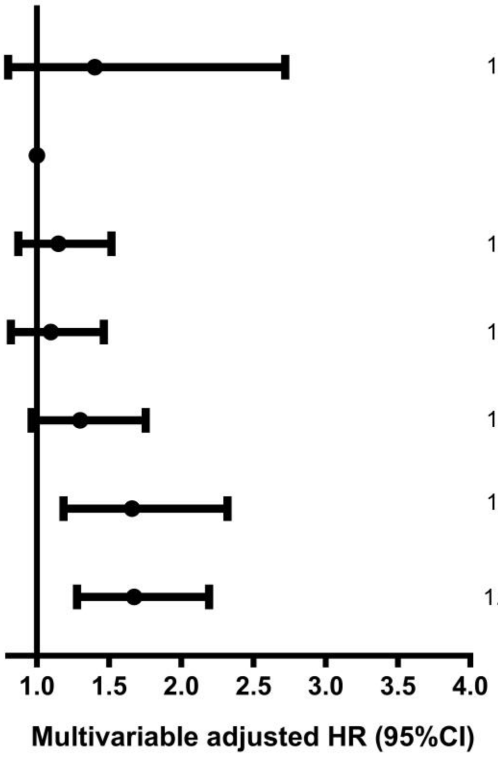

Supplementary Figure 10. All-cause mortality by NLR in individuals with prevalent or incident MPN

The multivariable-adjusted Cox regression analysis was adjusted for age, sex, hypertension, hyperlipidemia, CCI score, glucocorticoid use, CRP level, and education. The NLR 1-1.9 was used as a reference level.
All MPN combines both prevalent and incident MPN.
Correspond to Figures 3A & 3B – All-cause mortality by MPN.

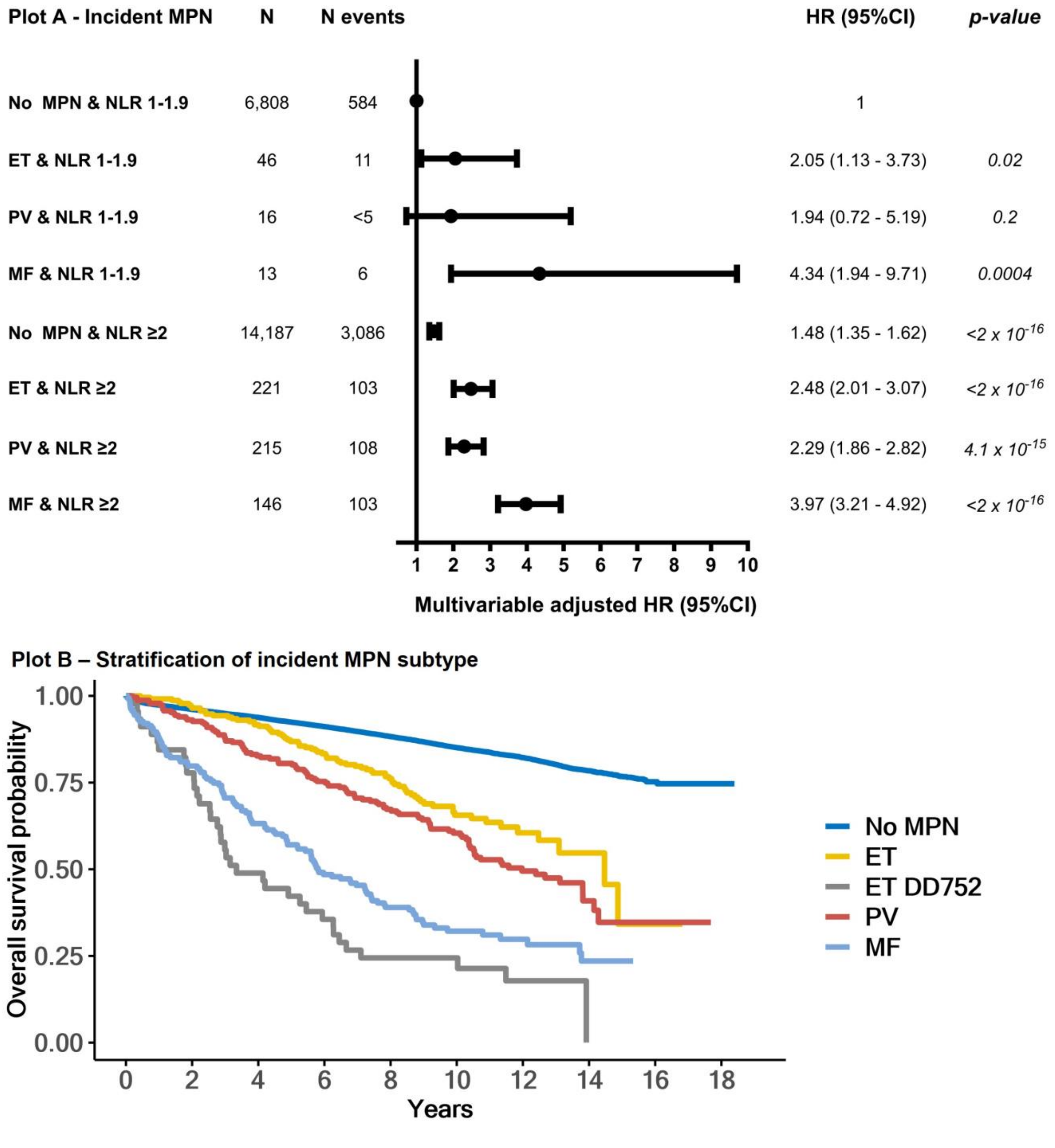

Supplementary Figure 11. All-cause mortality by NLR and MPN subtype

The multivariable-adjusted Cox-regression analysis was adjusted for age, sex, population origin, hypertension, hyperlipidemia, glucocorticoid use, CRP level, and education. NLR 1-1.9 without MPN was used as a reference level in Plot A. In Plot A – Incident MPN, the ET population is defined using both D752 and D473. Plot B stratify the ET population into D473 and D752. Clearly, D752 is not regular ET and must be misclassified. Correspond to Figure 4B – All-cause mortality by MPN subtype & NLR.

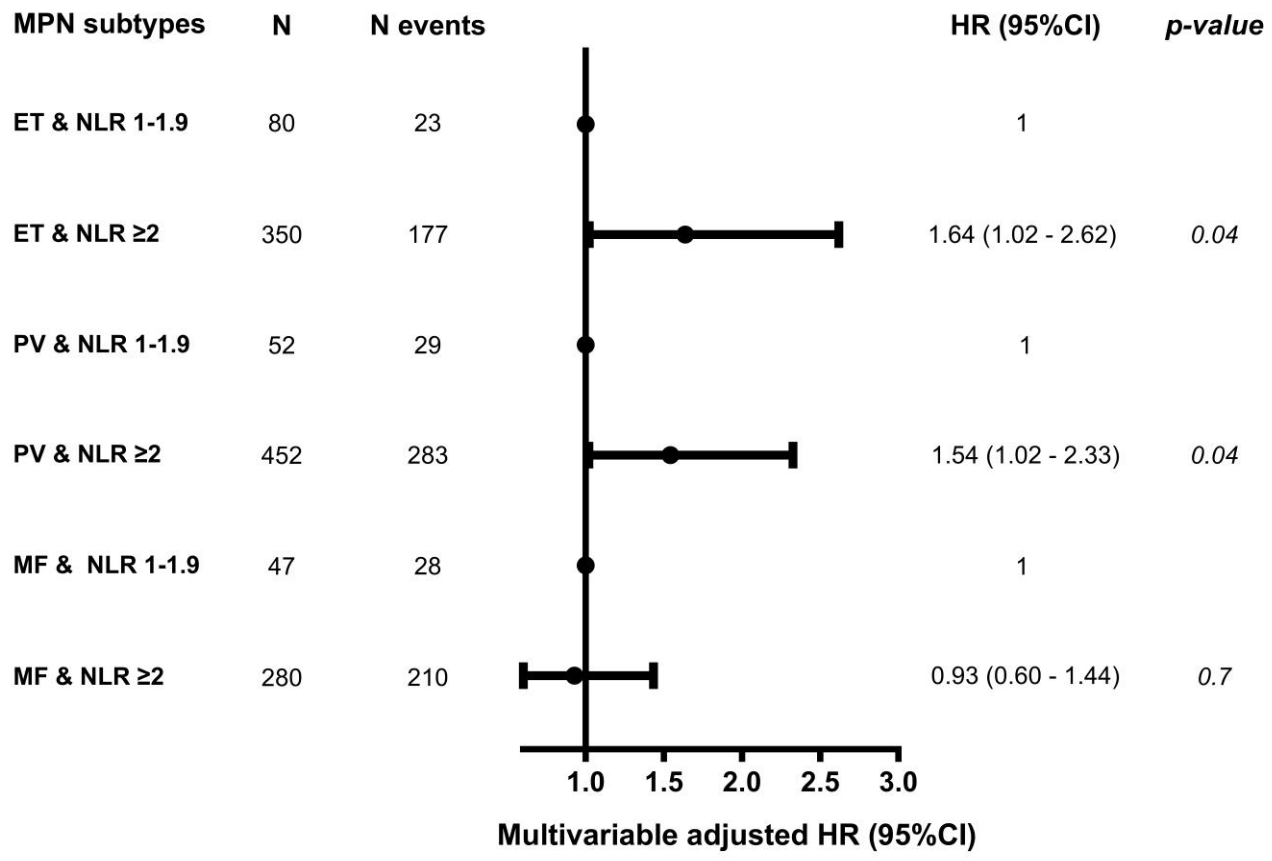

Supplementary Figure 12. All-cause mortality by NLR and MPN subtype in individuals with prevalent or incident MPN

The multivariable-adjusted Cox regression analysis was adjusted for age, sex, hypertension, hyperlipidemia, CCI score, glucocorticoid use, CRP level, and education. The NLR 1-1.9 was used as a reference level. MPN subtypes combine both prevalent and incident ET, PV, and MF.
Correspond to Figures 4A & 4B – All-cause mortality by MPN subtype.

Supplementary Table 12. Survival probabilities by NLR and CCI

	5-year survival	10-year survival	15-year survival	18-year survival
	**% (95%CI)**	**% (95%CI)**	**% (95%CI)**	**% (95%CI)**
**CCI-score**				
**0 CCI**				
**NLR <1**	98 (98 - 98)	95 (95 - 95)	91 (91 - 92)	88 (88 - 89)
**NLR 1-1.9**	98 (98 - 98)	95 (95 - 95)	91 (91 - 91)	88 (87 - 88)
**NLR 2-2.9**	96 (96 - 96)	92 (91 - 92)	86 (86 - 87)	83 (82 - 83)
**NLR 3-3.9**	93 (93 - 93)	87 (87 - 87)	81 (81 - 81)	77 (76 - 77)
**NLR 4-4.9**	90 (90 - 90)	83 (83 - 84)	78 (77 - 78)	74 (73 - 74)
**NLR 5-5.9**	88 (88 - 89)	81 (80 - 82)	75 (74 - 75)	71 (70 - 73)
**NLR ≥6**	84 (83 - 84)	75 (75 - 76)	69 (68 - 69)	66 (65 - 67)
**1-2 CCI**				
**NLR <1**	85 (84 - 86)	75 (74 - 76)	65 (63 - 66)	59 (57 - 61)
**NLR 1-1.9**	87 (87 - 88)	77 (76 - 77)	66 (66 - 67)	60 (59 - 61)
**NLR 2-2.9**	80 (80 - 80)	66 (66 - 67)	54 (54 - 55)	48 (47 - 49)
**NLR 3-3.9**	71 (71 - 72)	55 (55 - 56)	44 (44 - 45)	39 (38 - 40)
**NLR 4-4.9**	64 (64 - 65)	48 (47 - 49)	37 (36 - 38)	31 (29 - 32)
**NLR 5-5.9**	59 (58 - 61)	43 (42 - 44)	32 (31 - 33)	27 (25 - 29)
**NLR ≥6**	52 (51 - 52)	36 (35 - 36)	26 (26 - 27)	23 (22 - 24)
**≥3 CCI**				
**NLR <1**	60 (58 - 62)	42 (39 - 44)	31 (28 - 34)	26 (23 - 30)
**NLR 1-1.9**	65 (64 - 66)	46 (45 - 47)	34 (33 - 35)	29 (28 - 30)
**NLR 2-2.9**	54 (54 - 55)	34 (33 - 35)	23 (22 - 24)	18 (17 - 19)
**NLR 3-3.9**	44 (43 - 45)	26 (25 - 27)	16 (15 - 18)	12 (11 - 14)
**NLR 4-4.9**	37 (36 - 39)	20 (18 - 21)	12 (11 - 13)	7.5 (6.0 - 9.4)
**NLR 5-5.9**	32 (31 - 34)	16 (15 - 17)	9.5 (8.4 - 11)	5.9 (4.1 - 8.6)
**NLR ≥6**	25 (25 - 26)	12 (12 - 13)	7.3 (6.7 - 7.9)	5.6 (4.7 - 6.5)

Survival probabilities using 5-, 10-, 15-, and 18-year follow-up periods.
Correspond to Figure 5 – Mortality by comorbidity burden by NLR and CCI-score.

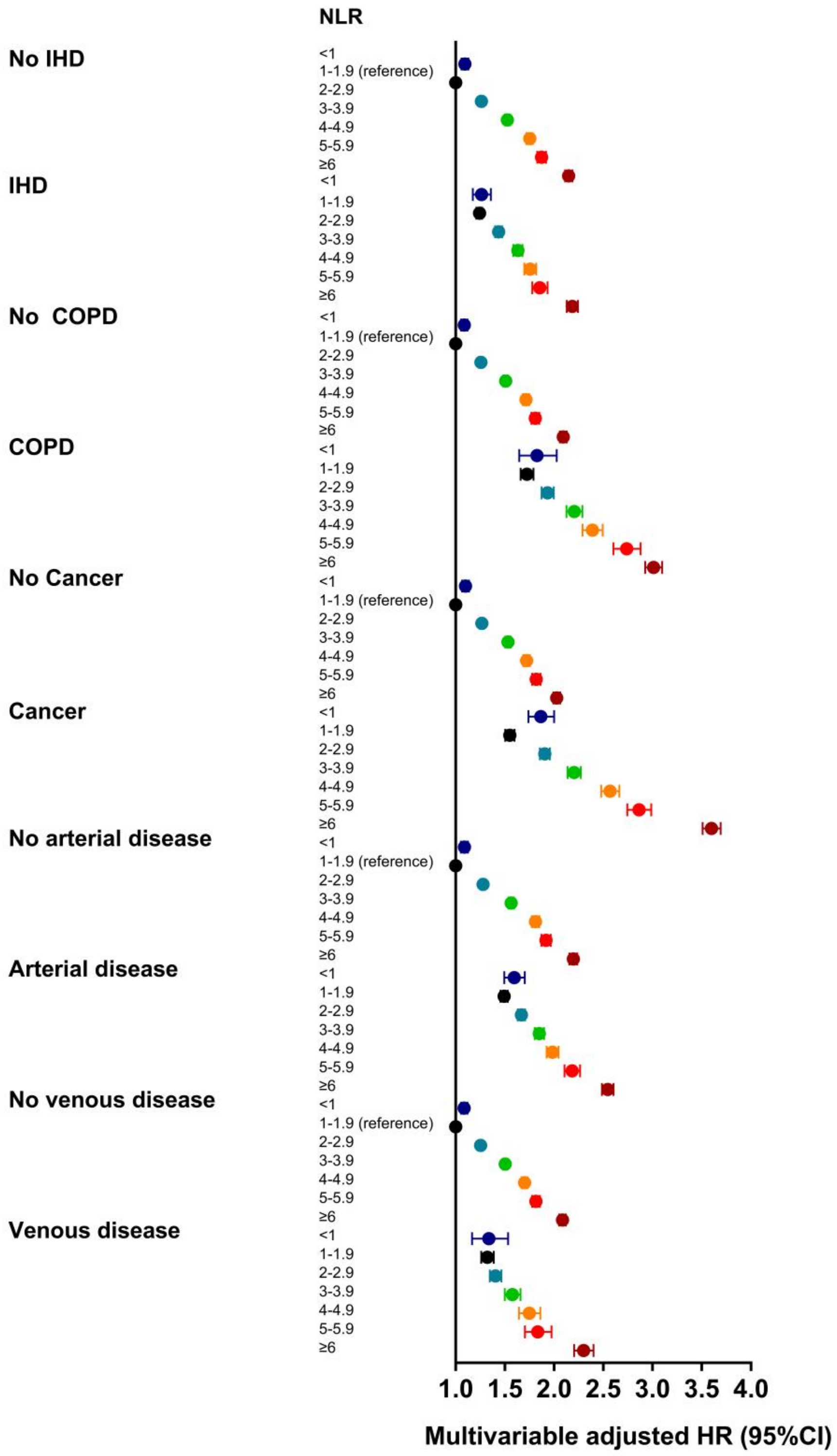

Supplementary Figure 13. Mortality by NLR and major prevalent disease categories

Cox proportional hazard regression models were used to obtain a multivariable-adjusted hazard ratio (HR). The Cox-regression was adjusted for potential confounders, including age, sex, population origin, hypertension, hyperlipidemia, CCI score (excluding the exposure variable IHD, COPD, cancer, arterial or venous disease), glucocorticoid use, CRP level, and education.
Abbreviations: HR: Hazard ratio derived from the Cox proportional hazard regression model. IHD: Ischemic heart disease. COPD: Chronic obstructive pulmonary disease. NLR: Neutrophil-to-lymphocyte ratio.

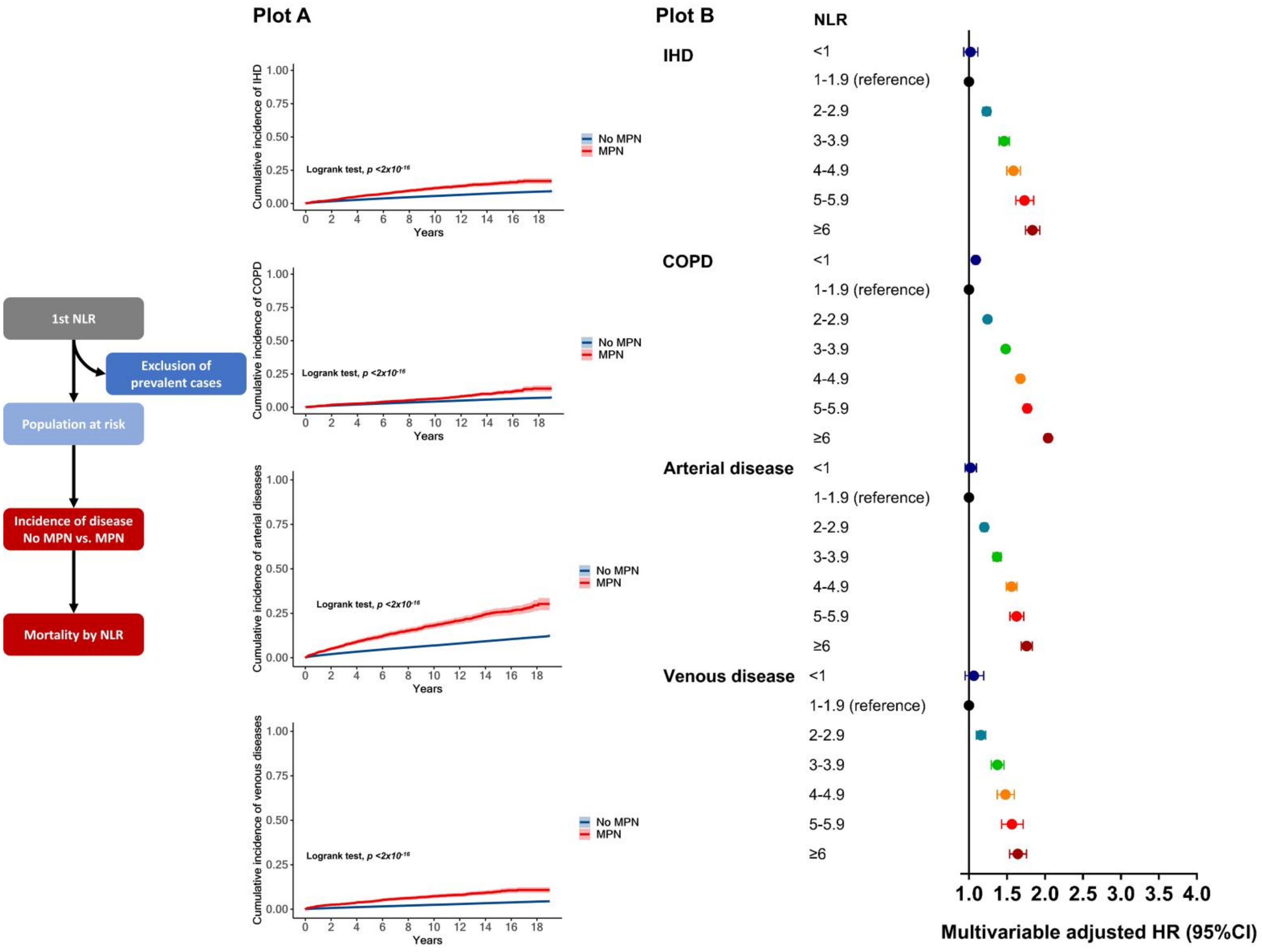

Supplementary Figure 14. Mortality by NLR and 1st occurring major incident disease

In plot A, the incidence of IHD, COPD, arterial, or venous diseases stratified by no MPN vs. combined prevalent and incident MPN. Time calculated since 1st NLR.
In plot B – All-Cause mortality by NLR among those with 1st occurring major incident disease.
Cox proportional hazard regression models were used to obtain a multivariable-adjusted hazard ratio (HR). The Cox-regression was adjusted for potential confounders, including age, sex, population origin, hypertension, hyperlipidemia, CCI score (excluding the exposure variable IHD, COPD, arterial or venous disease), glucocorticoid use, CRP level, and education.
Abbreviations: HR: Hazard ratio derived from the Cox proportional hazard regression model. IHD: Ischemic heart disease. COPD: Chronic obstructive pulmonary disease. MPN: Myeloproliferative neoplasms. Composite arterial disease was defined as either stroke, acute myocardial infarction, or peripheral artery disease of the lower extremity. The composite venous disease was defined as either deep venous thrombosis, pulmonary emboli, or splanchnic vein thrombosis. NLR: Neutrophil-to-lymphocyte ratio.

Supplementary Table 13. Comorbidities at the time of death by NLR

	NLR <1	NLR 1-1.9	NLR 2-2.9	NLR 3-3.9	NLR 4-4.9	NLR 5-5.9	NLR ≥6	
	N (%)	N (%)	N (%)	N (%)	N (%)	N (%)	N (%)	*p-value*
**Number of individuals**	5,270 (2.7)	42,341 (21.4)	49,118 (24.8)	31,054 (15.7)	18,919 (9.6)	12,163 (6.1)	38,937 (19.7)	
**Hypertension**	3,165 (60.1)	27,904 (65.9)	34,018 (69.3)	21,575 (69.5)	13,138 (69.4)	8,442 (69.4)	26,459 (68)	$< 1x10^{-4}$
**Hyperlipidemia**	1,501 (28.5)	14,142 (33.4)	15,892 (32.4)	9,438 (30.4)	5,495 (29)	3,466 (25.5)	9,975 (25.6)	$< 1x10^{-4}$
**IHD**	1,297 (24.6)	11,370 (26.9)	14,167 (28.8)	9,127 (29.4)	5,526 (29.2)	3,588 (29.5)	10,945 (28.1)	$< 1x10^{-4}$
**COPD**	946 (18)	8,059 (19)	10,208 (20.8)	6,585 (21.2)	4,158 (22)	2,771 (22.8)	9,172 (23.6)	$< 1x10^{-4}$
**Arterial disease**	1,771 (33.6)	15,737 (37.2)	19,422 (39.5)	12,750 (41.1)	7,868 (41.6)	5,078 (41.7)	15,902 (40.8)	$< 1x10^{-4}$
AMI	707 (13.4)	6,132 (14.5)	7,631 (15.5)	5,040 (16.2)	3,036 (16)	1,939 (15.9)	6,083 (15.6)	$< 1x10^{-4}$
Stroke	1,022 (19.4)	8,824 (20.8)	11,010 (22.4)	7,177 (23.1)	4,427 (23.4)	2,896 (23.8)	9,112 (23.4)	$< 1x10^{-4}$
TCI	338 (6.4)	3,135 (7.4)	3,841 (7.8)	2,489 (8.0)	1,440 (7.6)	1,018 (8.4)	2,868 (7.4)	$< 1x10^{-4}$
PAD	246 (4.7)	2,722 (6.4)	3,581 (7.3)	2,395 (7.7)	1,559 (8.2)	984 (8.1)	2,957 (7.6)	$< 1x10^{-4}$
**Venous disease**	546 (10.4)	4,632 (10.9)	5,366 (10.9)	3,379 (10.9)	1,993 (10.5)	1,370 (11.3)	3,950 (10.1)	0.0006
DVT	349 (6.6)	2,855 (6.7)	3,404 (6.9)	2,108 (6.8)	1,229 (6.5)	844 (6.9)	2,411 (6.2)	0.0008
PE	243 (4.6)	2,180 (5.1)	2,421 (4.9)	1,556 (5.0)	941 (5.0)	659 (5.4)	1,877 (4.8)	0.08
Splanchnic VT	16 (0.3)	94 (0.2)	94 (0.2)	57 (0.2)	31 (0.2)	19 (0.2)	79 (0.2)	0.3
**CCI-score**								
0	2,277 (43.2)	20,294 (47.9)	20,000 (40.7)	10,836 (34.9)	5,684 (30)	3,334 (27.4)	9,215 (23.7)	
1-2	1,973 (37.4)	15,862 (37.5)	20,066(40.9)	13,386 (43.1)	8,414 (44.5)	5,464 (44.9)	18,007 (46.2)	$< 1x10^{-4}$
≥3	1,020 (19.4)	6,185 (14.6)	9,052 (18.4)	6,832 (22)	4,821 (25.5)	3,365 (27.7)	11,715 (30.1)	

Hypertension and hyperlipidemia were defined by antihypertensive or lipid-lowering medication.
The date of death was used as the index date for the retrospective assessment of comorbidities.
Abbreviations: IHD: Ischemic heart Disease. COPD: Chronic obstructive pulmonary disease. AMI: Acute myocardial infarction. TCI: Transient cerebral ischemia. PAD: Peripheral artery disease of the lower extremity. DVT: Deep vein thrombosis. PE: Pulmonary emboli. Splanchnic VT: Splanchnic vein thrombosis. CCI-score: Charlson comorbidity index score.

Supplementary Table 14. Survival probabilities by MPN and CCI-score

	5-year survival	10-year survival	15-year survival	18-year survival
	**% (95%CI)**	**% (95%CI)**	**% (95%CI)**	**% (95%CI)**
**Plot A**				
**No MPN**				
**0 CCI**	95 (95 - 95)	91 (91 - 91)	86 (86 - 86)	83 (83 - 83)
**1-2 CCI**	75 (75 - 75)	61 (61 - 61)	51 (50 - 51)	45 (45 - 45)
**≥3 CCI**	45 (44 - 45)	27 (27 - 28)	18 (18 - 19)	15 (14 -15)
**Prevalent MPN**				
**0 CCI**	78 (73 - 83)	59 (53 - 65)	45 (38 - 52)	35 (25 - 48)
**1-2 CCI**	52 (46 - 59)	29 (24 - 36)	25 (19 - 32)	-
**≥3 CCI**	40 (33 - 50)	19 (14 - 28)	12 (6.7 - 20)	-
**Plot B**				
**No MPN**				
**0 CCI**	98 (98 – 98)	94 (94 – 95)	90 (90 – 91)	-
**1-2 CCI**	89 (88 – 90)	78 (77 – 79)	64 (62 – 66)	-
**≥3 CCI**	69 (67 – 72)	52 (49 – 54)	38 (35 – 42)	-
**Incident MPN**				
**0 CCI**	88 (84 – 91)	69 (63 – 74)	49 (39 – 61)	-
**1-2 CCI**	72 (67 – 78)	47 (41 – 54)	23 (13 – 40)	-
**≥3 CCI**	40 (31 – 52)	19 (12 – 30)	-	-

Survival probabilities using 5-, 10-, 15-, and 18-year follow-up periods.
For Plot B, we only calculated survival probability until the 15th year, given the shorter observation time.
Correspond to Figure 6 – Mortality by comorbidity burden by MPN and CCI-score.

Supplementary Table 15. Comorbidities at the time of death by MPN

	No MPN	MPN*	
	N (%)	N (%)	*p-value*
**Number of individuals**	196,796 (99.5)	1,006 (0.5)	
**Hypertension**	133,955 (68.1)	746 (74.2)	< $1x10^{-4}$
**Hyperlipidemia**	59,588 (30.3)	321 (31.9)	0.3
**IHD**	55,695 (28.3)	325 (32.3)	0.005
**COPD**	41,675 (21.2)	224 (22.3)	0.4
**Arterial disease**	78,032 (39.7)	496 (49.3)	< $1x10^{-4}$
AMI	30,399 (15.4)	169 (16.8)	0.3
Stroke	44,212 (22.5)	256 (25.4)	0.03
TCI	15,028 (7.6)	101 (10)	0.005
PAD	14,303 (7.3)	141(14)	< $1x10^{-4}$
**Venous disease**	21,056 (10.7)	180 (17.9)	< $1x10^{-4}$
DVT	13,084 (6.6)	116 (11.5)	< $1x10^{-4}$
PE	9,797 (5)	80 (8)	< $1x10^{-4}$
Splanchnic VT	380 (0.2)	10 (1)	< $1x10^{-4}$
**CCI-score**			
0	71,248 (36.2)	392 (39)	
1-2	82,766 (42.1)	406 (40.4)	0.2
≥3	42,782 (21.7)	208 (20.7)	

*Prevalent and incident MPN
Hypertension and hyperlipidemia were defined by antihypertensive or lipid-lowering medication. The date of death was used as the index date for the retrospective assessment of comorbidities.
Abbreviations: IHD: Ischemic heart disease. COPD: Chronic obstructive pulmonary disease. AMI: Acute myocardial infarction. TCI: Transient cerebral ischemia. PAD: Peripheral artery disease of the lower extremity. DVT: Deep vein thrombosis. PE: Pulmonary emboli. Splanchnic VT: Splanchnic vein thrombosis. CCI-score: Charlson comorbidity index score.

Supplementary Table 16. Baseline characteristics by the Triple-A risk score (1st NLR)

	Low risk		Intermediate-1 risk		Intermediate-2 risk		High risk		
	N	% / Mean(SD)	N	% / Mean(SD)	N	% / Mean(SD)	N	% / Mean(SD)	*p-value*
**Sex**									
Female	233,622	58.1	138,395	52.6	46,913	60.3	54,395	58.8	$<2.2x10^{-16}$
Male	168,172	41.9	124,901	47.4	30,888	39.7	38,144	41.2	
**Age**	401,794	35.3 (8.4)	263,296	58.4 (7.7)	77,801	75.9 (8.7)	92,539	80.5 (6.6)	$<2.2x10^{-16}$
**Education**									
Basic	69,010	17.2	74,423	28.3	28,072	36.1	30,240	32.7	
Upper secondary	28,236	7	9,351	3.6	1,228	1.6	1,218	1.3	
Vocational training	139,452	34.7	108,401	41.2	24,150	31	23,540	25.4	$<2.2x10^{-16}$
Bachelor	80,506	20	42,499	16.1	6,421	8.3	6,322	6.8	
Higher education	73,313	18.2	22,274	8.5	3,163	4,.1	3,829	4.1	
Unknown	11,277	2.8	6,348	2.4	14,767	19	27,390	29.6	
**Glucocorticoids**									
No use	398,591	99.2	259,406	98.5	75,750	97.4	89,204	96.4	
Early use	2,338	0.6	2,674	1	1,365	1.8	2,122	2.3	$<2.2x10^{-16}$
Late use	865	0.2	1,216	0.5	686	0.9	1,213	1.3	
**Comorbidities**									
Hypertension	21,734	5.4	84,456	32.1	44,250	56.9	58,079	62.8	$<2.2x10^{-16}$
Hyperlipidemia	6,024	1.5	35,476	13.5	14,833	19.1	15,701	17	$<2.2x10^{-16}$
IHD	4,707	1.2	22,837	8.7	14,069	18.1	19,310	20.9	$<2.2x10^{-16}$
COPD	2,262	0.6	10,378	3.9	6,609	8.5	10,463	11.3	$<2.2x10^{-16}$
Arterial disease	5,457	1.4	25,902	9.8	17,938	23.1	25,852	27.9	$<2.2x10^{-16}$
Venous disease	3,964	1	6,768	2.6	3,647	4.7	5,284	5.7	$<2.2x10^{-16}$
Cancer	5,676	1.4	18,520	7	10,870	14	16,544	17.9	$<2.2x10^{-16}$
CCI-score									
0	352,573	87.7	174,606	66.3	34,344	44.1	31,540	34.1	
1-2	44,924	11.2	70,143	26.6	30,678	39.4	40,625	43.9	$<2.2x10^{-16}$
≥3	4,297	1.1	18,547	7	12,779	16.4	20,374	22	
**Blood cell counts**									
Neutrophil ($x10^9/L$)	401,794	4.6 (2.3)	263,296	5 (2.9)	77,801	6 (3.5)	92,539	7 (4.5)	$<2.2x10^{-16}$
Lymphocyte ($x10^9/L$)	401,794	2.1 (1)	263,296	2.1 (1.9)	77,801	2.4 (4.1)	92,539	1.3 (2.8)	$<2.2x10^{-16}$
NLR	401,794	2.3 (1.4)	263,296	2.9 (2.9)	77,801	3.8 (5)	92,539	6.4 (6.2)	$<2.2x10^{-16}$
CRP-level									
No CRP	197,567	49.2	144,193	54.8	46,292	59.5	56,325	60.9	
CRP ≤10mg/L	165,994	41.3	89,443	34	20,629	26.5	17,765	19.2	$<2.2x10^{-16}$
CRP >10mg/L	38,233	9.5	29,660	11.3	10,880	14	18,449	19.9	

Early glucocorticoid use was defined as any redeemed prescription -15 prior to the first blood sample date.
Late glucocorticoid use was defined as any redeemed prescription -16 to -30 days prior to the first blood sample date.
Hypertension and hyperlipidemia were defined by antihypertensive or lipid-lowering medication.
The blood sample date was used as the index date for the retrospective assessment of comorbidities.
Abbreviations: CCI: Charlson comorbidity index score, NLR: Neutrophil-to-lymphocyte ratio. IHD: Ischemic heart disease. COPD: Chronic obstructive pulmonary disease. CRP: C-reactive protein.

Supplementary Table 17. CCI-score by the Triple-A risk score (1st NLR)

	Low risk	Intermediate-1 risk	Intermediate-2 risk	High risk	
	N (%)	N (%)	N (%)	N (%)	*p-value*
**Number of individuals**	401,794 (48.1)	263,296 (31.5)	77,801 (9.3)	92,539 (11.1)	
**Acute myocardial infarction**					
No	399,775 (99.5)	252,116 (95.8)	70,367 (90.4)	82,484 (89.1)	
Yes	2,019 (0.5)	11,180 (4.2)	7,434 (9.6)	10,055 (10.9)	$< 1x10^{-4}$
**Heart failure**					
No	400,860 (99.8)	257,686 (97.9)	71,870 (92.4)	81,637 (88.2)	
Yes	934 (0.2)	5,610 (2.1)	5,931 (7.6)	10,902 (11.8)	$< 1x10^{-4}$
**Peripheral vascular disease**					
No	400,568 (99.7)	256,784 (97.5)	72,702 (93.4)	85,551 (92.4)	
Yes	1,226 (0.3)	6,512 (2.5)	5,099 (6.6)	6,988 (7.6)	$< 1x10^{-4}$
**Cerebrovascular disease**					
No	397,281 (98.9)	246,869 (93.8)	65,894 (84.7)	74,380 (80.4)	
Yes	4,513 (1.1)	16,427 (6.2)	11,907 (15.3)	18,159 (19.6)	$< 1x10^{-4}$
**Dementia**					
No	401,683 (100.0)	262,062 (99.5)	74,844 (96.2)	86,775 (93.8)	
Yes	111 (0.0)	1,234 (0.5)	2,957 (3.8)	5,764 (6.2)	$< 1x10^{-4}$
**Chronic Pulmonary disease**					
No	384,312 (95.6)	246,041 (93.4)	69,641 (89.5)	80,201 (86.7)	
Yes	17,482 (4.4)	17,255 (6.6)	8,160 (10.5)	12,338 (13.3)	$< 1x10^{-4}$
**Rheumatic diseases**					
No	395,537 (98.4)	255,968 (97.2)	74,330 (95.5)	87,074 (94.1)	
Yes	6,257 (1.6)	7,328 (2.8)	3,471 (4.5)	5,465 (5.9)	$< 1x10^{-4}$
**Peptic ulcer disease**					
No	398,053 (99.1)	254,719 (96.7)	73,037 (93.9)	85,231 (92.1)	
Yes	3,741 (0.9)	8,577 (3.3)	4,764 (6.1)	7,308 (7.9)	$< 1x10^{-4}$
**Mild liver disease**					
No	398,407 (99.2)	258,280 (98.1)	76,770 (98.7)	91,391 (98.8)	
Yes	3,387 (0.8)	5,016 (1.9)	1,031 (1.3)	1,148 (1.2)	$< 1x10^{-4}$
**Severe liver disease**					
No	401,280 (99.9)	262,171 (99.6)	77,588 (99.7)	92,289 (99.7)	
Yes	514 (0.1)	1,125 (0.4)	213 (0.3)	250 (0.3)	$< 1x10^{-4}$
**Diabetes without end-organ damage**					
No	396,892 (98.8)	250,844 (95.3)	72,181 (92.8)	85,592 (92.5)	
Yes	4,902 (1.2)	12,452 (4.7)	5,620 (7.2)	6,947 (7.5)	$< 1x10^{-4}$
**Diabetes with end-organ damage**					
No	399,793 (99.5)	256,531 (97.4)	74,546 (95.8)	88,454 (95.6)	
Yes	2,001 (0.5)	6,765 (2.6)	3,255 (4.2)	4,085 (4.4)	$< 1x10^{-4}$
**Hemiplegia**					
No	400,828 (99.8)	262,476 (99.7)	77,530 (99.7)	92,194 (99.6)	
Yes	966 (0.2)	820 (0.3)	271 (0.3)	345 (0.4)	$< 1x10^{-4}$
**Moderate to severe renal disease**					
No	400,256 (99.6)	261,006 (99.1)	76,506 (98.3)	90,162 (97.4)	
Yes	1,538 (0.4)	2,290 (0.9)	1,295 (1.7)	2,377 (2.6)	$< 1x10^{-4}$
**Any tumor**					
No	395,244 (98.4)	238,840 (90.7)	65,815 (84.6)	74,770 (80.8)	
Yes	6,550 (1.6)	24,456 (9.3)	11,986 (15.4)	17,769 (19.2)	$< 1x10^{-4}$
**Metastatic solid tumor**					
No	401,040 (99.8)	260,307 (98.9)	76,501 (98.3)	90,555 (97.9)	
Yes	754 (0.2)	2,989 (1.1)	1,300 (1.7)	1,984 (2.1)	$< 1x10^{-4}$
**AIDS/HIV**					
No	401,271 (99.9)	263,051 (99.9)	77,773 (100.0)	92,525 (100.0)	
Yes	523 (0.1)	245 (0.1)	28 (0.0)	14 (0.0)	$< 1x10^{-4}$
**Leukemia**					
No	401,473 (99.9)	262,646 (99.8)	77,253 (99.3)	92,282 (99.7)	
Yes	321 (0.1)	650 (0.2)	548 (0.7)	257 (0.3)	$< 1x10^{-4}$
**Lymphoma**					
No	400,999 (99.8)	261,631 (99.4)	77,192 (99.2)	91,526 (98.9)	
Yes	795 (0.2)	1,665 (0.6)	609 (0.8)	1,013 (1.1)	$< 1x10^{-4}$

Each CCI-score component by the Triple-A risk score
P-value calculated by Pearson´s Chi-squared test.
The blood sample date was used as the index date for retrospective assessment of comorbidities.
Each CCI component includes ICD8 & ICD10 codes, as presented in Supplementary Table 2.
Abbreviations: NLR: Neutrophil-to-lymphocyte ratio.

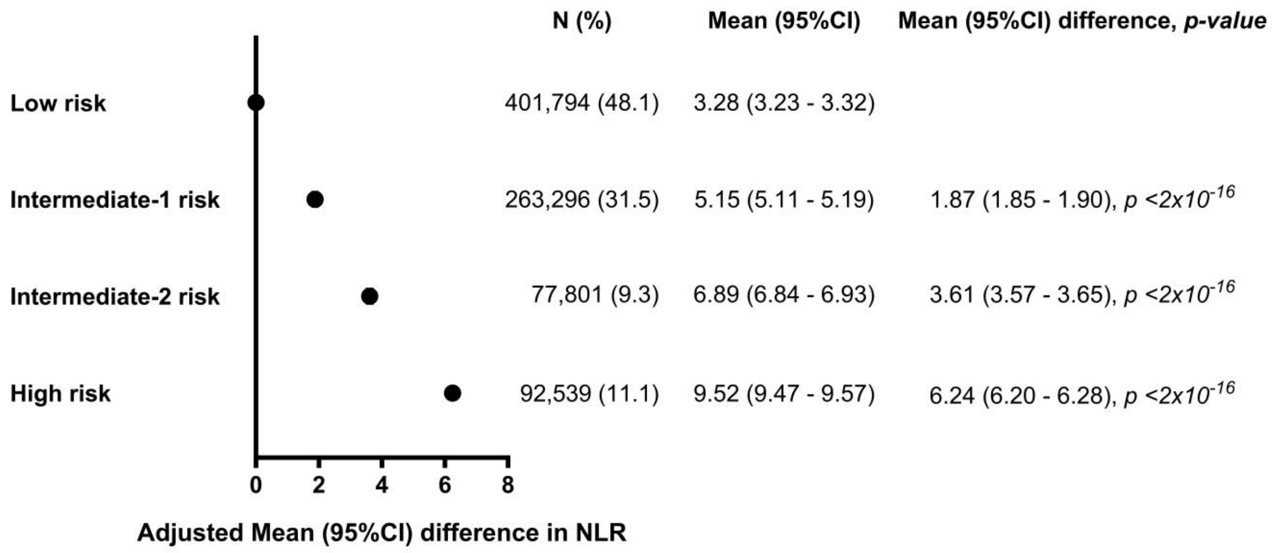

Supplementary Figure 15. Mean (95%CI) NLR by the Triple-A risk score

Adjusted mean (95%CI) in NLR by Triple-A risk score in the whole population. The multiple-adjusted linear regression analysis was adjusted for age, sex, population origin, hypertension, hyperlipidemia, CCI score, glucocorticoid use, CRP level, and education.
Abbreviations: NLR: Neutrophil-to-lymphocyte ratio. CCI: Charlson comorbidity index score

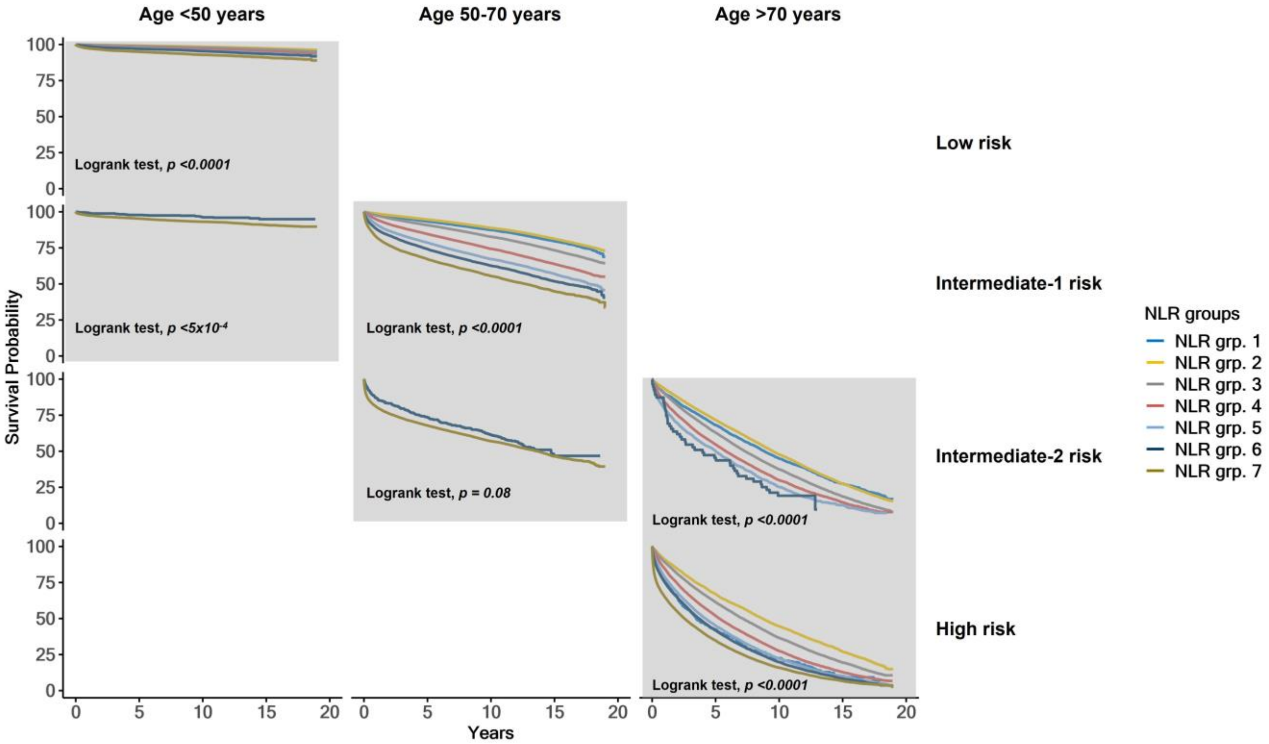

Supplementary Figure 16. Survival probability by NLR across the Triple-A risk score

Kaplan-Meier matrix for survival probability investigating NLR groups across the Triple-A on the whole population.
Gray-shaded areas within the Kaplan-Meier matrix represent the age categories used in the Triple-A.
Vertically, the Triple-A risk categories are Low risk, Intermediate-1 risk, Intermediate-2 risk, and High risk.
The Logrank test was used to compare the survival distribution between NLR groups for each matrix
Abbreviations: Triple-A risk score – integration of age, absolute lymphocyte count (ALC), and absolute neutrophil count (ANC). NLR: Neutrophil-to-lymphocyte ratio.

Supplementary Table 18. All-cause mortality by the Triple-A risk score, unadjusted

	Unadjusted model			
	**N**	**N events**	**HR (95%CI)**	***p-value***
**Whole population**				
**Low risk**	401,794	11,940	1	
**Intermediate-1 risk**	263,296	58,815	8 (8.20 – 8.53)	$<2x10^{-16}$
**Intermediate-2 risk**	77,801	51,180	36 (35.7 – 37.1)	$<2x10^{-16}$
**High risk**	92,539	75,867	62 (60.5 – 62.9)	$<2x10^{-16}$
**All MPN***				
**Low risk**	136	7	1	
**Intermediate-1 risk**	467	201	10 (4.71 – 21.28)	$2.1x10^{-9}$
**Intermediate-2 risk**	201	129	21 (9.67 – 44.36)	$6.2x10^{-15}$
**High risk**	479	423	44 (20.99 – 93.87)	$<2x10^{-16}$

All MPN: Includes both prevalent and incident MPN.
Abbreviations: Triple-A risk score – integration of age, absolute lymphocyte count (ALC), and absolute neutrophil count (ANC).

Supplementary Table 19. Comorbidities at the time of death by the Triple-A risk score

	Low risk	Intermediate-1 risk	Intermediate-2 risk	High risk	
	N (%)	N (%)	N (%)	N (%)	*p-value*
**Number of individuals**	11,940 (6)	58,815 (29.7)	51,180 (25.8)	75, 867 (38.4)	
**Hypertension**	3,938 (33)	36,164 (61.5)	38,072 (74.4)	56,527 (74.5)	$< 1x10^{-4}$
**Hyperlipidemia**	2,005 (16.8)	22,018 (37.4)	16,420 (32.1)	19,466 (25.7)	$< 1x10^{-4}$
**IHD**	1,222 (10.2)	14,313 (24.3)	16,362 (32)	24,123 (31.8)	$< 1x10^{-4}$
**COPD**	1,372 (11.5)	13,519 (23)	11,149 (21.8)	15,859 (20.9)	$< 1x10^{-4}$
**Arterial disease**	1,888 (15.8)	19,867 (33.8)	28,875 (44.7)	33,898 (44.7)	$< 1x10^{-4}$
AMI	642 (5.4)	7,908 (13.4)	9,081 (17.7)	12,937 (17.1)	$< 1x10^{-4}$
Stroke	1,092 (9.1)	10,840 (18.4)	12,993 (25.4)	19,543 (25.8)	$< 1x10^{-4}$
TCI	229 (1.9)	3,302 (5.6)	4,644 (9.1)	6,954 (9.2)	$< 1x10^{-4}$
PAD	286 (2.4)	4,232 (7.2)	4,174 (8.2)	5,752 (7.6)	$< 1x10^{-4}$
**Venous disease**	1,127 (9.4)	6,365 (10.8)	5,635 (11)	8,109 (10.7)	$< 1x10^{-4}$
DVT	705 (5.9)	3,828 (6.5)	3,572 (7)	5,095 (6.7)	$< 1x10^{-4}$
PE	519 (4.3)	3,030 (5.2)	2,605 (5.1)	3,723 (4.9)	0.001
Splanchnic VT	42 (0.4)	202 (0.3)	66 (0.1)	80 (0.1)	$< 1x10^{-4}$
**CCI-score**					
0	7,044 (59)	23,808 (40.5)	18,297 (35.8)	22,491 (29.6)	
1-2	3,569 (29.9)	23,227 (39.5)	21,960 (42.9)	34,416 (45.4)	$< 1x10^{-4}$
≥3	1,327 (11.1)	11,780 (20)	10,923 (21.3)	18,960 (25)	

Hypertension and hyperlipidemia were defined by antihypertensive or lipid-lowering medication.
The date of death was used as the index date for the retrospective assessment of comorbidities.
Abbreviations: IHD: Ischemic heart disease. COPD: Chronic obstructive pulmonary disease. AMI: Acute myocardial infarction. TCI: Transient cerebral ischemia. PAD: Peripheral artery disease of the lower extremity. DVT: Deep vein thrombosis. PE: Pulmonary emboli. Splanchnic VT: Splanchnic vein thrombosis. CCI-score: Charlson comorbidity index score.

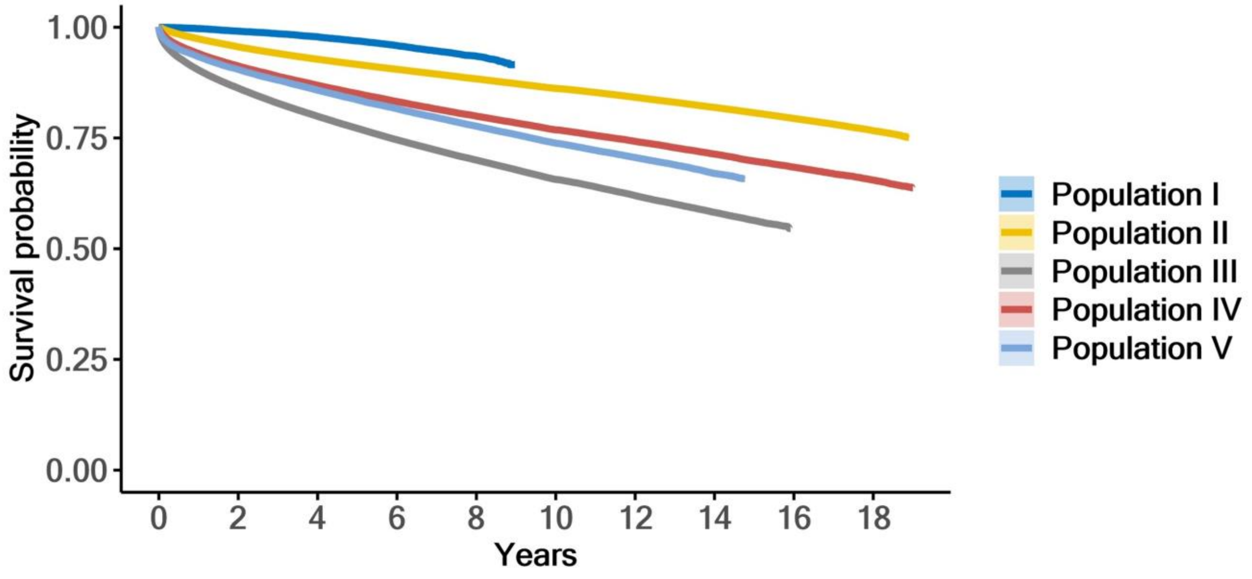

Observation time in years

	N	Median	Q1	Q3	Min	Max
**Population Origin**						
Population I	18,938	7.3	6.1	8.1	0.02	9
Population II	494,768	12.1	8.3	15.8	0	18.9
Population III	207,748	9.5	5.8	12.2	0	15.9
Population IV	69,367	12	8.1	15.9	0	19
Population V	44,609	13	9.4	13.9	0	14.8

Supplementary Figure 17. Survival probabilities by population origin with total observation time

Kaplan-Meier curve with survival probability (%) on the y-axis and years of follow-up since the first occurring blood sample on the x-axis.
The population was grouped into people I-V based on origin.
Population I: The General Suburban Population Study (GESUS)
Population II: LIMS from General Practitioners (GP)
Population III: LIMS from Capital Region Hospitals – Hospital cohort
Population IV: LIMS from Region Zealand Hospitals – Hospital cohort
Population V: LIMS from Region North Hospitals – Hospital cohort
